# Common Human Cancer Genes Discovered by Integrated Gene-Expression Analysis

**DOI:** 10.1371/journal.pone.0001149

**Published:** 2007-11-07

**Authors:** Yan Lu, Yijun Yi, Pengyuan Liu, Weidong Wen, Michael James, Daolong Wang, Ming You

**Affiliations:** Department of Surgery and the Alvin J. Siteman Cancer Center, Washington University School of Medicine, St. Louis, Missouri, United States of America; South African National Bioinformatics Institute, South Africa

## Abstract

**Background:**

Microarray technology enables a standardized, objective assessment of oncological diagnosis and prognosis. However, such studies are typically specific to certain cancer types, and the results have limited use due to inadequate validation in large patient cohorts. Discovery of genes commonly regulated in cancer may have an important implication in understanding the common molecular mechanism of cancer.

**Methods and Findings:**

We described an integrated gene-expression analysis of 2,186 samples from 39 studies to identify and validate a cancer type-independent gene signature that can identify cancer patients for a wide variety of human malignancies. The commonness of gene expression in 20 types of common cancer was assessed in 20 training datasets. The discriminative power of a signature defined by these common cancer genes was evaluated in the other 19 independent datasets including novel cancer types. QRT-PCR and tissue microarray were used to validate commonly regulated genes in multiple cancer types. We identified 187 genes dysregulated in nearly all cancerous tissue samples. The 187-gene signature can robustly predict cancer versus normal status for a wide variety of human malignancies with an overall accuracy of 92.6%. We further refined our signature to 28 genes confirmed by QRT-PCR. The refined signature still achieved 80% accuracy of classifying samples from mixed cancer types. This signature performs well in the prediction of novel cancer types that were not represented in training datasets. We also identified three biological pathways including glycolysis, cell cycle checkpoint II and plk3 pathways in which most genes are systematically up-regulated in many types of cancer.

**Conclusions:**

The identified signature has captured essential transcriptional features of neoplastic transformation and progression in general. These findings will help to elucidate the common molecular mechanism of cancer, and provide new insights into cancer diagnostics, prognostics and therapy.

## Introduction

Cancer is one of the leading causes of death in Western countries resulting in one of every four deaths. More than 100 types of cancer with different incidence have been diagnosed in various organs or tissues. Cancer is associated with multiple genetic and regulatory aberrations in the cell. To capture these abnormalities, DNA microarrays, which permit the simultaneous measurement of expression levels of tens of thousands of genes, have been increasingly utilized to characterize the global gene-expression profiles of tumor cells and matched normal cells of the same origin. Over the past years, the global gene-expression profiles of various cancers have been analyzed and many gene-expression signatures that are associated with cancer progression, prognosis and response to therapy have been described [1]–[19]. However, such studies are typically specific to certain tumors. The cancer type-specific signatures from these studies show little overlap in gene constitutions and biologically important pathways. Decades of research in molecular oncology have yielded few useful tumor-specific molecular markers, due to limitations with sample availability, identification, acquisition, integrity, and preparation [20]. Cancer is a highly heterogeneous disease, both morphologically and genetically. It remains a challenge to capture an essential, common transcriptional feature of neoplastic transformation and progression.

To extract maximum value from the recent accumulation of publicly available cancer gene-expression data, it is necessary to evaluate, integrate and inter-validate multiple datasets. Comprehensive analyses of a myriad of published datasets make it possible to find common cancer genes and essential functional consequences that are associated with tumor initiation and progression in general. Systematic characterization of expression changes in biological pathways among different types of cancer will eventually lead to a better understanding of which perturbations in the cell give rise to cancer. These findings will provide multiple clinical directions for cancer diagnostics, prognostics and therapy on the basis of the gene expression signature of patients. In the present study, we described an integrated gene-expression analysis of 2,186 samples from 39 different studies to identify and validate a cancer gene signature that is independent of tumor types and can identify cancer patients for a wide variety of human malignancies.

## Results

### Common gene expression changes in various cancer types

We first analyzed gene expression profiles of 1,223 human samples (343 normal tissues and 880 tumor tissues) from the training datasets 1–20 containing 20 different types of cancers (**Table S1**). The commonness of gene expression in these 20 cancer types was statistically assessed by permutation analyses (p<10^−5^). In total, 187 genes commonly affected in cancer were identified. Of these, 117 were up-regulated and 70 were down-regulated in nearly all cancerous tissue samples, regardless of their tissue of origin (**Table 1** **and** **2**). With the bioinformatics tool, FatiGO (http://fatigo.bioinfo.cnio.es), we found 142 out of 187 cancer genes were significantly associated with at least one Gene Ontology (GO) category. Several functional categories have been shown to be important for carcinogenesis and cancer progression (**Table S2**). For example, 11 genes (BFAR, CARD4, SPP1, SNCA, BAX, STAT1, CLU, GULP1, BID, CIDEA and PPP2R1B) control programmed cell death; 8 genes (TTK, RECK, BAX, STAT1, NME1, CCNB2, E2F3 and PPP2R1B) are involved in regulation of the cell cycle; 8 genes (TAP1, APOL2, SPP1, CLU, PSMB8, TAPBP, HLA-F and TNFSF13B) play roles in the immune response; and 6 genes (TTK, SPP1, NME1, NAP1L1, NPM1 and TNFSF13B) regulate cell proliferation. In addition, genes that are involved in protein transport, M phase cell cycle, secretory pathway and DNA repair are consistently up-regulated in a large majority of cancer types.

**Table 1:** Common up-regulated genes in human malignancies

UniGene	Gene symbol	N	Up #	Down #	UniGene	Gene symbol	N	Up #	Down #
Hs.159430	FNDC3B	11	10	0	Hs.239388	PAQR8	8	5	1
Hs.518201	DTX3L	8	7	0	Hs.592827	RBAK	8	5	1
Hs.530899	LOC162073	8	7	0	Hs.525157	TNFSF13B	8	5	1
Hs.15159	CKLF	11	9	1	Hs.126774	DTL	13	8	0
Hs.474150	BID	16	13	0	Hs.385913	ANP32E	13	8	1
Hs.7753	CALU	15	12	0	Hs.532968	DKFZp762E1312	13	8	1
Hs.418795	GLT25D1	10	8	0	Hs.372429	PDIA6	13	8	1
Hs.435556	BFAR	12	9	0	Hs.233952	PSMA7	13	8	1
Hs.459362	PRC1	12	9	1	Hs.533770	SLC38A1	13	8	1
Hs.521800	C8orf76	8	6	0	Hs.489284	ARPC1B	18	11	0
Hs.209561	KIAA1715	8	6	0	Hs.497788	EPRS	18	11	0
Hs.585011	C1orf96	8	6	1	Hs.79110	NCL	18	11	0
Hs.403933	FBXO32	8	6	1	Hs.251531	PSMA4	18	11	0
Hs.368853	AYTL2	15	11	1	Hs.429180	EIF2S2	18	11	1
Hs.511093	NUSAP1	11	8	0	Hs.465885	ILF3	18	11	1
Hs.370895	RPN2	14	10	0	Hs.169840	TTK	18	11	1
Hs.180062	PSMB8	17	12	0	Hs.489365	AP1S1	15	9	1
Hs.444600	BOLA2	10	7	0	Hs.256639	PPIH	15	9	1
Hs.445890	CNIH4	13	9	0	Hs.14559	CEP55	10	6	1
Hs.534392	KDELR3	13	9	0	Hs.308613	MTERFD1	10	6	1
Hs.632191	XTP3TPA	13	9	0	Hs.21331	ZWILCH	10	6	1
Hs.387567	ACLY	19	13	1	Hs.524599	NAP1L1	17	10	1
Hs.533282	NONO	18	12	0	Hs.78771	PGK1	17	10	2
Hs.83753	SNRPB	18	12	0	Hs.512380	PLEKHB2	12	7	1
Hs.471441	PSMB2	18	12	1	Hs.352018	TAP1	19	11	1
Hs.482497	TNPO1	18	12	1	Hs.194698	CCNB2	14	8	1
Hs.370937	TAPBP	15	10	0	Hs.153357	PLOD3	14	8	1
Hs.126941	FAM49B	12	8	0	Hs.471200	NRP2	14	8	2
Hs.408629	KDELC1	12	8	0	Hs.250822	AURKA	16	9	1
Hs.497384	IPO9	12	8	1	Hs.75528	GNL2	16	9	1
Hs.8752	TMEM4	12	8	1	Hs.1197	HSPE1	16	9	1
Hs.195642	C17orf27	9	6	0	Hs.202672	DNMT1	18	10	1
Hs.358997	TTL	9	6	0	Hs.433670	FTL	18	10	1
Hs.1600	CCT5	20	13	0	Hs.519972	HLA-F	18	10	1
Hs.269408	E2F3	17	11	0	Hs.520210	KDELR2	18	10	1
Hs.234027	ZBTB12	17	11	1	Hs.405153	CARD4	11	6	1
Hs.520205	EIF2AK1	14	9	0	Hs.477700	DBR1	11	6	1
Hs.89545	PSMB4	14	9	0	Hs.14468	FLJ11286	11	6	1
Hs.449415	EIF2C2	14	9	1	Hs.516077	FLJ14668	11	6	1
Hs.409065	FEN1	14	9	1	Hs.494337	GOLPH2	11	6	1
Hs.313	SPP1	14	9	1	Hs.371036	NOX4	11	6	1
Hs.525135	FARP1	14	9	2	Hs.438683	SLAMF8	11	6	1
Hs.524390	K-ALPHA-1	11	7	0	Hs.520714	SNX10	11	6	1
Hs.432360	SCNM1	11	7	0	Hs.159428	BAX	13	7	1
Hs.172028	ADAM10	19	12	0	Hs.311609	DDX39	13	7	1
Hs.381189	CBX3	19	12	0	Hs.463035	FKBP10	13	7	1
Hs.522257	HNRPK	19	12	0	Hs.438695	FKBP11	13	7	1
Hs.470943	STAT1	19	12	0	Hs.515255	LSM4	13	7	1
Hs.118638	NME1	19	12	1	Hs.552585	MORC2	13	7	1
Hs.519452	NPM1	19	12	1	Hs.43666	PTP4A3	13	7	1
Hs.506748	HDGF	16	10	0	Hs.369440	SFXN1	13	7	1
Hs.386283	ADAM12	16	10	2	Hs.517155	TMEPAI	13	7	1
Hs.474740	APOL2	8	5	0	Hs.631580	UBA2	13	7	1
Hs.552608	C1orf58	8	5	0	Hs.463465	UTP18	13	7	1
Hs.470654	CDCA7	8	5	0	Hs.492974	WISP1	13	7	1
Hs.179838	FMNL3	8	5	0	Hs.113876	WHSC1	13	7	2
Hs.143818	GEMIN6	8	5	0	Hs.494614	BAT2D1	15	8	2
Hs.6459	GPR172A	8	5	0	Hs.166463	HNRPU	19	10	2
Hs.133294	IQGAP3	8	5	0					

N: number of studies (types of cancer) which have available expression data on a tested gene.

Up # or down #: number of cancer types whose expression of the tested gene is up- or down-regulated.

All these genes are significantly consistently up-regulated (P<10^−5^) in a large majority of cancer types.

**Table 2:** Common down-regulated genes in human malignancies

UniGene	Gene symbol	N	Up #	Down #	UniGene	Gene symbol	N	Up #	Down #
Hs.401835	TCEAL2	10	0	8	Hs.306083	LOC91689	8	0	5
Hs.58351	ABCA8	13	0	10	Hs.160953	P53AIP1	8	0	5
Hs.525205	NDRG2	12	0	9	Hs.211252	SLC24A3	8	0	5
Hs.524085	USP2	12	0	9	Hs.163079	TUBAL3	8	0	5
Hs.172755	BRP44L	11	0	8	Hs.389171	PINK1	13	0	8
Hs.22242	ECHDC3	11	0	8	Hs.470887	GULP1	13	1	8
Hs.196952	HLF	19	1	13	Hs.490981	MSRA	13	1	8
Hs.496587	CHRDL1	12	0	8	Hs.476092	CLEC3B	18	0	11
Hs.476319	ECHDC2	12	0	8	Hs.386502	FMO4	18	0	11
Hs.409352	FLJ20701	12	0	8	Hs.137367	ANK2	18	1	11
Hs.103253	PLIN	12	0	8	Hs.212088	EPHX2	18	1	11
Hs.293970	ALDH6A1	18	1	12	Hs.157818	KCNAB1	18	1	11
Hs.390729	ERBB4	17	0	11	Hs.163924	NR3C2	18	1	11
Hs.553502	RORA	17	0	11	Hs.269128	PPP2R1B	18	1	11
Hs.388918	RECK	14	0	9	Hs.40582	CDC14B	15	1	9
Hs.216226	SYNGR1	14	0	9	Hs.438867	FLJ20489	10	1	6
Hs.506357	FAM107A	14	1	9	Hs.224008	FEZ1	17	1	10
Hs.476454	ABHD6	11	0	7	Hs.443789	C6orf60	12	1	7
Hs.519694	C5orf4	11	0	7	Hs.475319	LRRFIP2	12	1	7
Hs.528385	DHRS4	11	0	7	Hs.514713	MPPE1	12	1	7
Hs.47288	TRPM3	11	0	7	Hs.183153	ARL4D	19	1	11
Hs.420830	HIF3A	11	1	7	Hs.642660	C10orf116	19	1	11
Hs.511265	SEMA6D	11	1	7	Hs.495912	DMD	19	1	11
Hs.436657	CLU	19	1	12	Hs.503126	SHANK2	14	1	8
Hs.78482	PALM	16	0	10	Hs.481342	SORBS2	14	1	8
Hs.82318	WASF3	16	0	10	Hs.169441	MAGI1	16	1	9
Hs.268869	ADHFE1	8	0	5	Hs.75652	GSTM5	18	1	10
Hs.34494	AGXT2	8	0	5	Hs.405156	PPAP2B	18	1	10
Hs.249129	CIDEA	8	0	5	Hs.271771	SNCA	18	1	10
Hs.302754	EFCBP1	8	0	5	Hs.181855	CASC5	9	1	5
Hs.521953	EFHC2	8	0	5	Hs.506458	ANKS1B	11	1	6
Hs.200100	Ells1	8	0	5	Hs.445885	KIAA1217	11	1	6
Hs.479703	FLJ21511	8	0	5	Hs.643583	DKFZp667G2110	13	1	7
Hs.500750	HPSE2	8	0	5	Hs.406787	FBXO3	13	1	7
Hs.380929	LDHD	8	0	5	Hs.431498	FOXP1	13	1	7

All these genes are significantly consistently down-regulated (P <10^−5^) in a large majority of cancer types.

### Validation of common cancer genes by QRT-PCR and TMA

**Figure 1:**
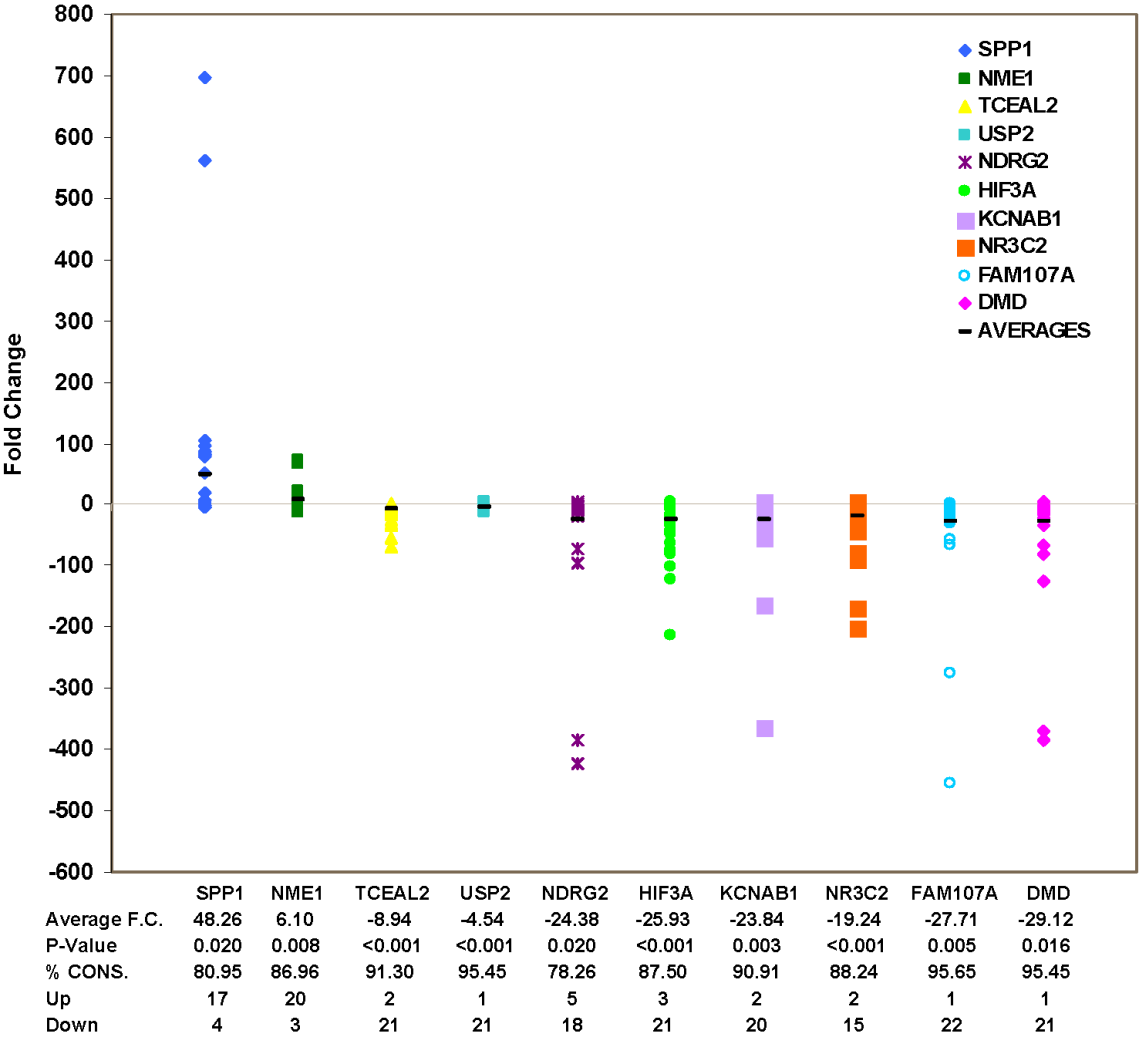
QRT-PCR analysis for the selected top ten scoring genes in expanded samples. Fold differences over matched normal controls were plotted for 21 to 24 tumors from all tested tissues including breast, lung, prostate, colorectal and cervical tumors in duplicate, providing 42 to 48 data points per gene. The average fold change, p value, consistency of regulation tendency, and numbers of tissues with over or under-expression were also listed.

To validate the microarray gene expression results from the integrated gene-expression analysis, the relative expression levels of 32 of the 187 cancer genes were determined by QRT-PCR analysis using completely independent samples from three each of breast, lung, prostate, colon and cervical cancer and their matched normal tissue. We confirmed the expression results for most of these selected genes (fold change >1.5, p≤0.05 and consistency >60%) (**Table 3**). The top 10 genes with absolute fold change >4, p≤0.05 and consistency >85% were further confirmed using another 18 matched tumor and normal samples from breast, lung and cervical cancer patients. In the expanded analysis, the expression levels of these 10 genes were still significantly different with absolute fold change >4, p≤0.05 and consistency >85%, except genes SPP1 and NDRG2, which exhibited slightly decreased consistency (**Figure 1**).

**Table 3:** QRT-PCR analysis of selected common cancer genes in initial screens

Gene****	Fold Change*						P-value**	Consistency*** (%)
	Breast	Lung	Prostate	Colon	Cervical	Average		
**DMD**	−447.7	−127.3	−4.9	−7.9	−10.4	−119.7	0.0242	93.33
HLF	−368.7	−2.6	−2.4	−10.5	−12.1	−90.3	0.0816	84.62
ERBB4	−352.7	−4.6	0.2	−2.4	−24.6	−85.1	0.0713	64.29
SEMA6D	−198.6	−81.7	−0.4	−6.7	5.4	−60.8	0.0432	78.57
PLIN	−170.1	3.6	0.1	−9.1	−3.0	−41.2	0.0609	69.23
ABCA8	−2041.8	−56.0	0.5	−14.3	−42.2	−40.9	0.0008	80.00
**NDRG2**	−161.8	−4.7	−1.2	−5.1	−3.3	−37.6	0.0318	85.71
**FAM107A**	−16.4	−24.9	−3.0	−6.5	−131.3	−36.4	0.0186	100.00
**KCNAB1**	−16.3	−97.4	−8.1	−8.7	−16.8	−32.1	0.0165	92.31
**NR3C2**	−71.4	−29.6	−1.7	−5.4	−23.6	−26.6	0.0036	85.71
CLU	−49.1	−4.3	−2.0	−3.7	−14.8	−14.8	0.0215	78.57
**HIF3A**	−23.3	−18.7	−3.2	−7.3	−17.7	−14.1	0.0000	86.67
**TCEAL2**	−29.5	−1.7	−3.9	−13.1	−8.6	−10.7	0.0005	86.67
ALDH6A1	−7.2	0.7	1.4	−2.0	−22.8	−7.8	0.0119	75.00
RORA	−14.6	−7.5	1.2	−2.1	−1.3	−5.5	0.0041	81.82
EPHX2	−13.7	−0.3	0.6	−5.2	−6.2	−5.0	0.0050	60.00
**USP2**	−3.4	−0.2	−4.6	−8.8	−4.6	−4.6	0.0000	92.86
PPP2R1B	−18.2	−2.0	−0.5	−0.6	1.7	−3.9	0.0226	66.67
ANK2	−4.2	−0.8	−2.6	−5.0	−6.7	−3.9	0.0000	85.71
SYNGR1	−7.6	−1.9	−1.5	−5.2	−0.7	−3.4	0.0000	92.31
BRP44L	−6.9	−3.5	9.8	−12.9	−2.7	−3.3	0.0000	86.67
CDC14B	−6.3	−5.7	−1.7	0.6	0.4	−2.8	0.0014	76.92
RECK	−4.5	−2.7	0.2	−0.5	−0.9	−1.7	0.0000	73.33
PGK1	0.1	0.9	0.6	0.1	12.3	3.2	0.1323	61.54
PRC1	1.6	6.2	0.8	−0.3	8.7	3.2	0.0369	64.29
BID	0.9	3.7	3.5	−0.2	9.1	3.4	0.0003	80.00
CCT5	0.3	8.6	2.5	−0.6	6.7	3.7	0.0256	71.43
FEN1	0.0	5.7	1.8	−0.5	15.6	4.5	0.0407	73.33
**NME1**	0.6	25.4	3.6	2.2	6.3	7.6	0.0197	86.67
NUSAP1	2.7	10.2	5.7	0.6	35.8	11.0	0.0042	73.33
ADAM12	−5.2	4.9	−3.1	23.9	39.7	12.0	0.0084	53.33
**SPP1**	1.6	53.9	2.4	40.7	232.9	70.8	0.0117	85.71

* Fold change: negative and positive values indicate an increase and decrease in expression level of tumor tissues compared with normal tissues for each gene, respectively.

** P-value: one tailed Z-test comparing expression difference between normal and tumor groups.

*** Consistency: the percentage of paired samples used for QRT-PCR having the same regulation tendency in microarray data.

**** Genes marked bold are top ten scoring genes chosen for QRT-PCR analysis in expanded samples.

**Figure 2:**
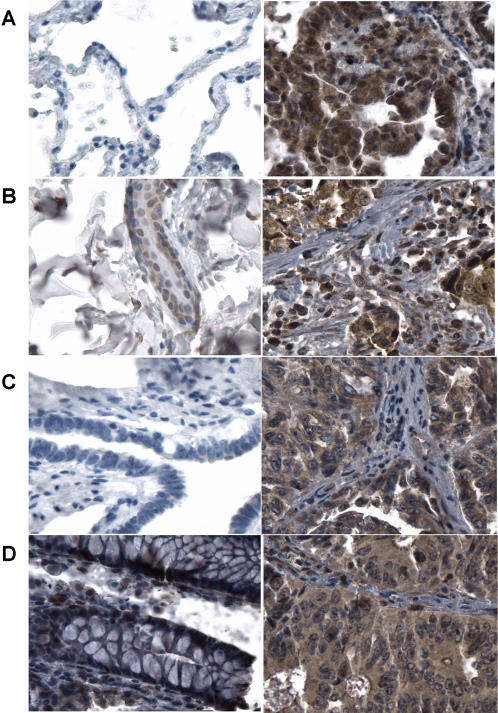
Immunostaining analysis of SPP1 expression in normal and tumor tissues. SPP1 positive staining presents in cytoplasm and nuclear of tumor cells in lung cancer (A, right), breast cancer (B, right), ovary cancer (C, right), and Colon cancer (D, right) while negative staining in normal lung (A, left), and ovary (C, left), weak staining in normal breast (B, left), and colon (D, left).

**Figure 3:**
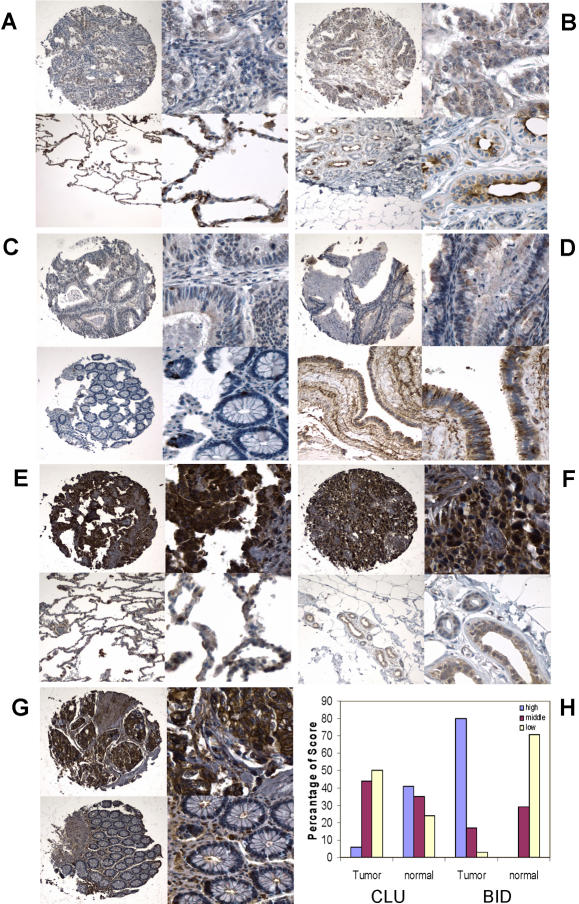
CLU and BID immunostaining in tumor and normal tissues. Positive cytoplasmic staining of CLU presents on both tumor and normal cells in lung, breast, colon, and ovary tissue (A to D). CLU expression decreased in lung cancer (A, upper level) in comparison with normal lung (A, lower level). Both normal breast (B, lower level) and ovary (D, lower level) show middle to strong staining in cytoplasm, and middle to weak staining in breast (B, upper level) and ovarian cancer (D, upper level). Much less positive clusterin staining present in both colon tumor (C, upper level) and normal tissues (C, lower level). BID shows strong cytoplasmic and nuclear staining in lung cancer (E, upper level) and weak nuclear staining in normal lung epithelium (E, lower level). Increased strong nuclear and cytoplasmic staining is seen in breast tumor (F, upper level) when compared with normal breast tissue (F, lower level). In colon cancer, BID shows strong cytoplasmic and nuclear staining in tumor cells (G, upper level), while less positive BID staining was found in normal colon tissue (G, lower level). Semiquantitative analysis of CLU and BID immunoreaction in tumors and normal tissues (H). Most of samples from different types of cancer have strong, high percentage of BID immunoreaction, and normal tissues only show middle to low level staining; while CLU tends to down-regulated in tumors (H). High = score 10–12, middle = score 6–9, and low = score 1–5. Left column in each panel is at low power (100X) and right column in each panel is at high power (400X).

Tissue microarray analysis (TMA) was also performed for three randomly picked common cancer genes (SPP1, BID and CLU) to determine if mRNA changes were correlated with changes in protein expression in cancer patients. The tissue microarray contains 200 tumor samples with 50 samples from each of four cancer types (colon, breast, ovarian, and lung). Analysis of SPP1 protein expression in tumor and normal tissues indicated that SPP1 is present in the cytoplasm and nucleus of cells. Most samples from colon adenocarcinoma, breast adenocarcinoma, ovary adenocarcinoma, and lung cancer showed intermediate to strong cytoplasmic SPP1 staining in tumor cells, but the staining in normal tissues was much weaker. The average scores for tumor and normal tissues are 11.1±1.8 and 1.8±1.5 in colon adenocarcinoma (p = 0.0005), 10.9±1.7 and 4.5±3.8 in breast adenocarcinoma (p = 0.043), 11.7±1.0 and 3.3±4.2 in ovary adenocarcinoma (p = 0.028), and 9.0±2.2 and 3.5±2.0 in lung cancer (p = 0.0005), respectively (**Figure 2** **and Figure S1**). Positive cytoplasmic staining of clusterin (CLU) was present on both tumor and normal cells in lung, breast, and ovary tissue. However, as compared with normal tissues, the clusterin decreased in lung cancer (5.6±2.1 *vs.* 10.8±1.6, p = 0.005), breast cancer (7.1±2.7 *vs.* 10.5±1.7, p = 0.017), and ovarian cancer (6.8±3.0 *vs.* 10.5±1.7, p = 0.011) (**Figure 3A, B and D**). Colon cancer showed much less positive CLU staining than other types of tumor and there was no significant difference between colon tumor and normal colon tissues (**Figure 3C**). BID protein was significantly upregulated in colon cancer, lung cancer and breast cancer, but not in ovarian cancer (data not shown). The average scores of immunoreactive staining in tumor and normal tissues are 11.2±1.9 *vs.* 2.0±1.4 (p = 0.00015), 10.4±2.2 *vs.* 2.5±2.2 (p = 0.0002) and 11.5±1.4 *vs.* 6.5±1.9 (p = 0.010) for these three cancer types, respectively (**Figure 3E–G**). Semiquantitative analysis indicates that most of samples from different types of cancer have strong, high percentage of BID immunoreaction, while normal tissues only show low to medium level of staining; CLU tends to be down-regulated in tumors (**Figure 3H**). The results demonstrate that protein level is largely consistent with the mRNA expression of these three genes.

### Common cancer pathways in various cancer types

We further surveyed a listing of 1,687 biological pathways which include metabolic pathways, protein interaction networks, signal transduction pathways, and gene regulatory networks to examine if several genes within a specific pathway act in a cumulative manner to influence neoplastic transformation and progression. The richness of significantly differentially expressed genes in a given pathway was again evaluated by 100,000 permutation tests in the training datasets. Pathway analysis showed that significant differentially expressed genes (p<0.01) were mostly enriched in the glycolysis pathway, cell cycle checkpoint II pathway and plk3 pathway, which included 24, 10 and 10 genes, respectively (p<10^−5^). Interestingly, most of the genes involved in these three pathways are up-regulated in a large majority of cancerous tissues as compared with normal tissues (**Figure S2**), suggesting the prevalence of gene hyperactivation and amplification in human malignancies.

### Confirmation of the common gene expression pattern in independent datasets

Next, we determined to validate our gene expression signature and see if we could distinguish cancer samples from normal samples in completely independent datasets. The discriminative power of the 187-gene expression signature in normal and tumor samples was tested by clustering analysis using oligonucleotide gene expression data obtained from 19 completely independent datasets. Datasets 21 to 38, used for validation, were comprised of 211 normal and 492 tumor tissues from 14 different cancer types. In most of these 18 datasets, samples were classified into two groups, one comprising of most normal samples and another for most tumor samples, based on our 187 gene signature (**Figure S3**). The overall accuracy of correct classification is, on average, 92.64% ranging from 78% to 100% (**Table 4**). It should be noted that dataset 30 and 31 are slightly different from the other datasets due to heterogeneity of cancer samples. All the samples in these two datasets were classified into two big groups: one containing tumor samples only; the other group containing both tumors and normals, which can be clearly distinguished as two subgroups. Specifically, in dataset 30, eight myeloma cell lines formed one group with inclusion of one plasma cell leukemia (PCL); in the other group, eight normal plasma cell samples and eight samples from patients with multiple myeloma (MM) or PCL were clearly subdivided. In dataset 31, six metastatic prostate cancer samples were grouped together, while normal tissues and primary prostate cancers were in the other group, with six normal tissues and one primary prostate cancer in one subgroup, and six primary prostate cancers in the other subgroup. In clustering analysis for tumor samples of different subtypes with gene expression data, it is not unusual that some tumor subgroups are closer to the normal group, but are clearly distinguished from the normal group.

**Table 4:** Confirmation of the common gene expression pattern in independent datasets

Dataset	Cancer types	NN	NT	NE	Accuracy (%)
21	Colon cancer	12	48	4	93.33
22	Esophageal adenocarcinomas	15	19	0	100.00
23	Gastric Cancer	8	22	1	96.67
24	Glioblastoma	4	31	4	88.57
25	Head neck hypopharyngeal cancer	4	34	8	78.95
26	Lung cancer	30	57	5	94.25
27	Lung cancer	5	5	0	100.00
28	Lung cancer	19	20	0	100.00
29	Lymphoma	6	21	0	100.00
30	Myeloma	8	17	0	100.00
31	Prostate cancer	6	13	1	94.74
32	Prostate cancer	41	71	19	83.04
33	Testicular germ cell tumors	14	23	1	97.30
34	Testicular germ cell tumors	3	20	4	82.60
35	Thyroid carcinoma	7	7	2	85.71
36	Mesothelioma	9	40	4	91.84
37	Uterine Leiomyomas	5	5	1	90.00
38	Soft Tissue Sarcoma	15	39	1	98.15
39	Multiclass cancer	81	180	39	85.06
Sum	/	292	672	94	92.64

NN: number of normal samples; NT: number of tumor samples; NE: number of errors, that is, number of samples that were falsely clustered; Accuracy (%): NE/(NN+NT).

**Figure 4:**
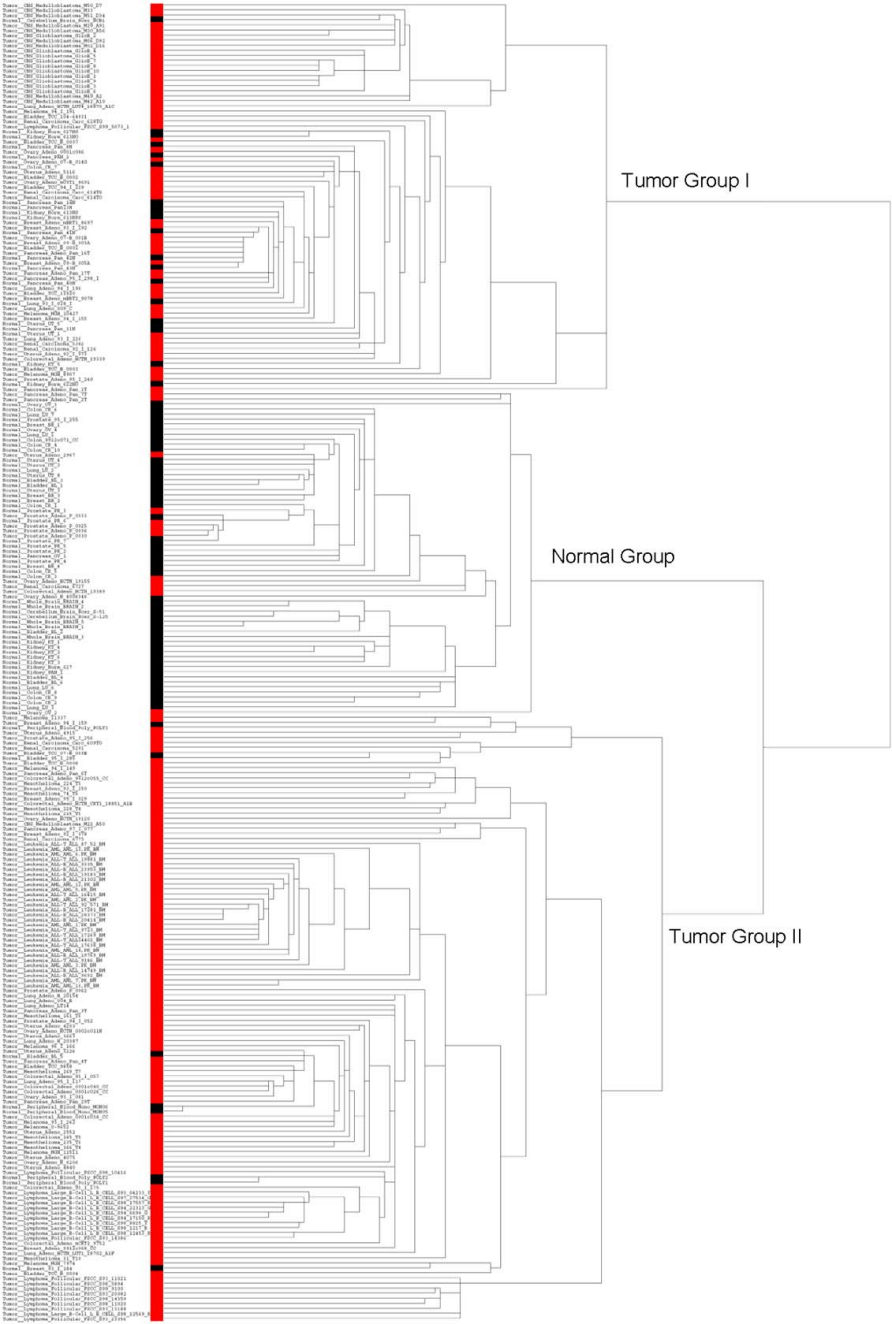
Hierarchical clustering of gene-expression profiles for 187 common cancer genes in dataset 39 with mixed cancer types. Normal tissues were marked black and tumor tissues were marked red. Samples are clustered into three groups: tumor group I composing of 57 tumors and 20 normal tissues, normal group composing of 11 tumors and 53 normal tissues, and tumor group II composing of 112 tumor and 8 normal tissues. All of central nervous system cancer and most of pancreatic adenocarcinoma were classified into tumor group I, while all of leukemia and most of lymphomas were classified into tumor group II. The accuracy of classification is 85%.

Dataset 39 included 180 tumor samples, spanning 14 common tumor types, and 81 normal tissue samples. Among 14 tumor types, uterine adenocarcinoma, leukemia and pleural mesothelioma were not present in the 20 training datasets. In clustering analyses, samples are clustered into three groups: tumor group I composing of 57 tumors and 20 normal tissues, normal group composing of 11 tumors and 53 normal tissues, and tumor group II composing of 112 tumor and 8 normal tissues (**Figure 4**). The accuracy of classification is 85%. All of central nervous system cancer and most of pancreatic adenocarcinoma were classified into tumor group I, while all of leukemia and most of lymphomas were classified into tumor group II. Notably, the 187-gene signature performs well (81–100%) in classifying new cancer types (such as uterine adenocarcinoma, leukemia and pleural mesothelioma). It is also worth noting that there only ∼31–60% of genes from this 187 gene signature that were used in clustering analyses in each of validation datasets due to the specificity of platforms, sample availability and missing values in microarray experiments. Furthermore, the set of genes used for clustering analyses are in part different among validation datasets, depending on the availability of gene-expression data in a specific study. This demonstrated the robustness, utility and ubiquity of our gene signature. Lastly, we also attempted to classify these 261 samples using the expression profiles of 28 common cancer genes confirmed by the QRT-PCR analysis with fold change >3 and consistency >60%. The refined 28-gene signature still achieved ∼80% accuracy of classification (Figure S4). It should be noted that dataset 39 used an old microarray system of Affymetrix FL 6800 gene chip with a total of 7,289 probes. The numbers of probes used in the above two clustering analyses for the dataset 39 were 72 and 19, corresponding to 59 and 15 genes, respectively.

## Discussion

DNA microarray-based gene-expression classification enables a standardized, objective assessment of oncological diagnosis and prognosis and provides complementary information to current clinical protocols [20]. However, such studies are typically specific to certain types of cancer, and the obtained expression profiles have limited use due to inadequate validation in large patient cohorts. In this study, we identified a gene signature for molecular cancer classification through an integrative gene-expression analysis of 20 different types of common cancer. This signature contains 187 genes whose aberrant expression was observed in nearly all cancerous tissue samples, regardless of their tissue of origin. To illustrate the utility and robustness of this signature, we determined its discriminative power on another 19 completely independent datasets. The accuracy of classification is about 92.6% by using this common cancer signature. Interestingly, a different subset of genes that account for 31–60% of the 187-gene signature can rigorously identify cancer patients for a wide variety of human malignancies. More importantly, this signature also performs well in the prediction of novel cancer types that were not represented in the integrative analysis in training datasets. This confirms that the identified signature is cancer type-independent and has captured certain of the essential transcriptional features of neoplastic transformation and progression in general. However, it remains unknown whether all of these genes in our signature are involved in the development of cancer. Some of them may be an indication of something going on in the body that is accompanying the disease process; while others are genes *pe se* that promote tumorigenesis and cancer progression. We also compared our signature with two other signatures in independent datasets that were previously used in those studies [21], [22]. The overall accuracy of correct classification using our signature is, on average, 95% ranging from 90% to 100%; while the overall accuracy of Rhode's and Xu's is 89% and 93%, respectively (**Table S3**). The two previous signatures were determined either from the same set of genes in a single microarray platform [21] or common genes among different platforms [22]. The analyzed genes only represent a subset of genes on the genome (about 25%) and were highly over-represented in their signatures; while the other genes that are not presented in the analyzed platform or are not common among platforms were missed in their signatures. In the present study, the proposed method for determining gene signature is straightforward and independent of different microarray platforms (for example various uncommercial cDNA chips and Affymetrix chips). Therefore, we can utilize information of all the genes in a specific microarray study. Our study also highlights the importance of the large sample size in microarray analyses for identifying and validating prognostic signatures. In this study, we pooled a total of 2,186 samples from 39 independent microarray studies for classifier discovery and validation. The results from this large-scale integrative gene-expression analysis should be more robust and reliable than each of potentially under-powered individual studies.

We also identified several common pathways where altered expression of several genes act in a common pathway to influence tumor development. These pathways include the glycolysis, cell cycle checkpoint II and plk3 pathways. We found that many of the genes within each of pathways were up-regulated in various types of tumor tissues as compared with normal tissues. The perturbation of expression of multiple genes within these pathways may be a common characteristic of neoplastic transformation and progression in malignant tumors. Therapeutic manipulation of these pathways may provide a universal strategy for treatment of many types of cancer. For example, cancer cells often generate energy through glycolytic fermentation rather than oxidative phosphorylation. It is possible that the lack of oxidative phosphorylation limits the production of proapoptotic superoxide. Three enzymes of the 187 gene profile, TPI, PGK1, and ENO1, which are involved in the glycolytic pathway, were also found to be significantly overexpressed in the HER-2/neu-positive breast tumors [23]. Overexpression of these enzymes may well relate to the increased requirements of both energy and protein synthesis/degradation pathways in the rapidly growing tumors. This pathway was proposed to be significant in tumorigenesis more than 70 years by Warburg [24].

The genes identified in our signature could be the prime targets of cancer therapy and prevention, since they are dysregulated in many types of cancer. Characterization of these common genes should provide opportunities for elucidating certain of the more general mechanisms of cancer initiation and progression. Cancer gene therapy classically involves delivery of tumor suppressor, apoptosis-inducing or suicide genes directly into tumor cells. Arrest of tumor cell proliferation is the ultimate objective of anticancer therapy. Interestingly, in our data, the identified common genes that are involved in regulation of cell proliferation are all up-regulated in different types of tumor tissues (**Table S2**). These genes include TTK, SPP1, NME1, NAP1L1, NPM1 and TNFSF13B. Osteopontin (SPP1) is a gene that regulates cell proliferation. Many studies have shown that SPP1 is highly expressed in several malignancies. Abundant secretion of SPP1 acts as a marker for breast and prostrate cancer, osteosarcoma, glioblastoma, squamous cell carcinoma and melanoma [25]. Cells from SPP1 knockout mice show impaired colony formation in soft agar and slower tumor growth *in vivo* in comparison with tumors in wild-type mice [26]. In our QRT-PCR analysis, SPP1 was overexpressed in 18 out of 22 samples from five different types of tumor tissues (**Figure 1**). The tissue microarray analysis further demonstrated that this increased mRNA expression level of SPP1 was significantly correlated with protein level in cancer patients (**Figure 2**). Thus, SPP1 may be a promising common target of cancer therapy and prevention.

BH3-interacting domain death agonist (BID) and clusterin (CLU) are two other potential therapeutic targets that are involved in programmed cell death. BID contains only the BH3 domain, which is required for its interaction with the Bcl-2 family proteins and for its pro-death activity. BID is susceptible to proteolytic cleavage by caspases, calpains, Granzyme B and cathepsins [27]. BID is important to cell death mediated by these proteases and thus is the sentinel to protease-mediated death signals [28]. Protease-cleaved BID is able to induce multiple mitochondrial dysfunctions, including the release of the inter-membrane space proteins, cristae reorganization, depolarization, permeability transition and generation of reactive oxygen species. Thus BID is a molecular bridge linking various peripheral death pathways to the central mitochondria pathway. Recent studies further indicated that BID may function as more than just a proapoptotic killer molecule. BID not only promotes cell cycle progression into S phase but also involves the maintenance of genomic stability by engaging at mitotic checkpoints [27]. This protein has diverse functions that are important to both the life and death of the cell. A recent study showed that BID increased in brain tumor, gliomas, prostate cancer, ovarian cancer and colon cancer [29]. CLU is a sulphated glycoprotein, implicated in various cell functions involved in carcinogenesis and tumor progression, including cell cycle regulation, cell adhesion, DNA repair and apoptosis. Several studies show greatly reduced expression of CLU in tumors compared with normal tissue, including testicular tumor, von Hippel-Lindau (pVHL)-defective renal tumor, esophageal squamous cell carcinoma[30]–[34]. The reduction in the overall CLU level appears because the CLU positive stromal compartments of the normal mucosa are lost in tumor [35]. CLU plays a negative role in epithelial cell proliferation and lack of CLU increases the susceptibility to tumorigenesis after carcinogenic challenge. The under-expression of CLU was immediately apparent in highly malignant MD PR317 prostate adenocarcinoma cells using laser microdissection technique and serial analysis of gene expression [36]. Both our QRT-PCR and tissue microarray analyses confirmed the upregulation of BID and downregulation of CLU in most of cancerous tissues (**Figure 3**).

Stem cells are the very earliest cells of the embryo that divide and differentiate to form mature organs and tissues. Small numbers of normal stem cells persist into adulthood and function to maintain and repair healthy tissues. It has been recently established that, like normal tissues, human tumors are initiated and maintained by stem cells. Cancer stem cells exist as a minority population within the tumor and share many genetic and biologic characteristics of normal stem cells. Some genes overexpressed in cancer tissues identified in our signature were found highly expressed in embryonic stem cells. For example, EPRS, NPM1, STAT1 and LSM4 are higher expressed in human embryonic stem cell lines compared with human universal RNA [37], [38]. CCNB1, FBXO2, NME2, SNRPF, DDX21, SLC38A4, PSMA2, PSMA3 and AP1S2 are also higher expressed in human embryonic stem cell lines [37], [38], members of these gene families such as CCNB2, FBXO32, NME1, SNRPB, DDX39, SLC38A1, PSMA4, PSMA7 and AP1S1 are observed in our signature. Particularly, two genes in our signature, DNMT1 and TAPBP, are listed on SuperArray GEArray S Series Human Stem Cell Gene Array, which is designed to profile the expression of genes known to be important for the identification, growth and differentiation of stem cells (Catalog number HS-601.2, Superarray, Frederick, MD; http://www.superarray.com/home.php). Our gene signature also includes several genes related to tissue development, such as regulation of developmental process (FNDC3B, SPP1 and TTL), embryonic development (ADAM10) and organ development (NCL, SPP1, SFXN1, BAX, ADAM12, NRP2 and NME1) (http://fatigo.bioinfo.cnio.es).

In summary, we defined a cancer-type-independent gene signature predictive of cancer status for a wide variety of human malignancies. This signature has captured the essential transcriptional transition of normal cell behavior to uncontrolled cell growth in malignant tumors and thus has significant implications in cancer diagnostics, prognostics and therapy. These genes should prove applicable to not only understand the common molecular mechanism of cancer, and cancer diagnosis, but also serve as potential molecular targets as well.

## Materials and Methods

### Data collection and processing

Microarray datasets were obtained from public databases. Data were of two general types, dual channel ratio data corresponding to spotted cDNA microarrays and single channel intensity data corresponding to Affymetrix microarrays. Thirty nine studies had 634 normal and 1,552 cancer samples in total (**Table S1**). All these previous microarray studies were originally designed for the identification of differentially expressed genes between normal and malignant tumor tissues for that specific type of cancer. Pathology reports were the basis for classify the normal and tumor tissue, and benign and malignant tumors in these studies. Datasets 1–20 which represent 20 different common cancer types such as bladder, breast, colon, endometrial, kidney, liver, lung, melanoma, lymphoma, pancreatic, prostate and thyroid cancer were used to identify common cancer genes and pathways, and datasets 21–39 were used for extensive validation. The chosen training datasets were normally larger than validation datasets except several very recently released datasets. All of the expression values were base-two log transformed. To facilitate multi-study analysis, Unigene cluster ID and gene names were assigned to all of the cDNA clones and Affymetrix probes based on the NCBI Unigene Build 198 (http://www.ncbi.nlm.nih.gov/entrez/query.fcgidbunigene).

### Detection of differentially expressed genes

We used two-sample permutation *t* test for identifying differentially expressed genes (DEGs), which was implemented in R package permax (http://www.r-project.org/), for each of the datasets 1–20. To obtain the robust results, 10,000 permutations were performed to calculate an empirical p value in the analysis of DEGs in each type of cancer.

### Common cancer biomarkers

To identify common biomarkers in different types of cancer, we used a permutation procedure to examine if DEGs (p<0.01) are statistically consistently up-regulated or down-regulated in different types of cancer. Specifically, we first determined how often a DEG is consistently up-regulated or down-regulated in different types of cancer in the original datasets. Then, we reshuffled cancer status (normal and tumor) and created 100,000 replicates for each type of cancer. In each replicate, we conducted the analysis of DEGs as described in the section “*Detection of differentially expressed genes*”, and record the number of cancer types in which a DEG is consistently up-regulated or down-regulated. Finally, the probability for observing a common biomarker in different types of cancer by random chance is calculated as,
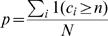
where *N* is the number of permutations, *n* is the number of cancer types in which a DEG is consistently up-regulated or down-regulated in the original datasets, and *c_i_* is the number of cancer types in which a DEG is consistently up-regulated or down-regulated in *i*-th permutation, *i* = 1,2,….*N*.

### Pathway analysis

Sets of genes that act in concert to carry out a specific function were also identified in different types of cancer. Gene sets we used are listed as c2 for curated gene sets in the Molecular Signature Database (MSigDB, http://www.broad.mit.edu/gsea/msigdb/msigdb_index.html). We also employed permutation analysis to examine if DEGs (p<0.01) are statistically significantly enriched in a given pathway. Specifically, we first record the number of DEGs in each of 1,687 pathways in original datasets. Then, we randomly reshuffled cancer status to create 100,000 replicates. In each replicate, we conducted the analysis of DEGs as described in the section “*Detection of differentially expressed genes*” and record the number of DEGs. The probability for observing the richness of DEGs in a given pathway by chance is calculated as,
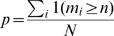
where *N* is the number of permutations, *n* is the number of DEGs observed in a given pathway in the original dataset, and *m_i_* is the number of DEGs observed in each randomly permuted replicate, *i* = 1,2,….20. This analysis was performed separately in each type of cancer.

### Clustering analysis

Hierarchical clustering based on centered Pearson correlation coefficient algorithm and average linkage method was used to show the expression patterns of common cancer genes in datasets 21 to 39. The datasets were normalized by standardizing each row (gene) to mean 0 and variance 1. The clustering analysis was performed by using CLUSTER and TREEVIEW software (http://rana.lbl.gov/EisenSoftware.htm). Classification accuracy for each of these datasets was calculated.

### QRT-PCR Analysis

Using 15 pairs of tumor and matched adjacent normal tissues from five cancer types (breast, lung, prostate, colon-recta and cervical cancer), three pairs for each cancer type, the relative expressions of 32 randomly chosen common cancer genes from the identified signature gene set were initially screened by QRT-PCR analysis, as described in a previous study [39]. These frozen tissues were acquired from Tissue Procurement Core at Washington University Siteman Cancer Center (St. Louis, Missouri, United States). Primers for the QRT-PCR analysis (**Table S4**) were designed using Primer Express software version 2.0 (Applied Biosystems, Foster City, CA). Amplification of each target gene was performed with SYBR Green master mix in BIO-RAD Single Color Real-Time PCR Detection system according to the manufacture protocols. The control gene β-Actin and target genes were amplified with equal efficiencies. The method for assessing if two amplicons have the same efficiency is to look at how ΔC_T_ (C_T,target_–C_T,β-Actin, _where C_T _is cycle number at which the fluorescence signal exceeds background) varies with template dilution, which is described in detail elsewhere [40]. The fold change of gene expression in normal tissues relative to tumor tissues was calculated as 2^−ΔΔCT^ (ΔΔCT = ΔC_T normal_–ΔC_T tumor_). One tailed Z-test was performed to determine statistical significance between normal and tumor groups. The average fold change of each gene and the consistency of the regulation tendency with the microarray data were also calculated. According to these three characteristics, the ten highest scoring genes were selected for further QRT-PCR confirmation using 18 matched tumor and normal samples from breast, lung and cervical cancer patients, three pairs for each cancer type.

### Tissue microarray

Tissue microarray (TMA) slides were purchased from the NCI Tissue Array Research Program (http://ccr.cancer.gov/tech_initiatives/tarp/). All samples were formalin-fixed, paraffin-embedded tissues. Limited demographic and pathology information was available at the NCI website (http://ccr.cancer.gov/tech_initiatives/tarp). The TMA slides contained four tumor types including colon adenocarcinoma, breast adenocarcinoma, ovary adenocarcinoma, lung cancer, and normal tissues, each from a distinct patient. The normal tissues were not paired with the tumor tissues on the slides. The tissue of origin for all samples was confirmed by experienced surgical pathologists. Each TMA slide contained 200 tissue samples of 0.6 mm and was ready for use in immunohistochemistry, but only among which there were limited normal tissues. In order to have enough normal tissue samples for comparison, we obtained additional normal tissue slides from Tissue Procurment Core at Washington University in St. Louis School of Medicine according to the approved protocol by Washington University in St Louis Human Studies Committee, such that the total number of normal tissues was 65. All slides were deparaffinized and rehydrated before antigen retrieval which was applied in microwave for 20 minutes with citrate buffer, pH 6.0. After blocking in 10% of normal goat serum in PBS, all primary antibodies were incubated overnight at 4°**C**, including SPP1 (Novocastra Laboratories, Newcastle, UK, clone 15G12, dilution 1∶100), BID (BD Tranduction Laboratories, San Jose, CA, clone 7, dilution 1∶200) and CLU (Upstate Biotech, Lake Placid, NY, clone 41D, dilution 1∶1000). The appropriate secondary biotinylated IgG (1∶500) was used, followed by ABC method (Vectastain ABC Elite Kit, Vector Lab, Burlingame, CA) and diaminobenzidine (DAB) (Sigma, St. Louis, MO) was used as chromogen. For negative control, the primary antibody was omitted with normal serum. The percentage of positive cancer cells was scored on a semiquantitative scale as 0 (0%), 1 (1–20%), 2 (20–50%), 3 (50–75%) and 4 (over 75%). Intensity was scored as 1 (weak), 2 (middle) and 3 (strong). Results were calculated by multiplying the score of percentage of positive cells (P) by the intensity (I). The maximum score is 12. The evaluation of immunostaining results was performed independently by two investigators. Student's t test was used to assess the significance of expression difference from normal and tumor tissues.

## Supporting Information

Figure S1 The immunostaining images of SPP1 in various cancer tissue microarray. The sections from normal tissues are shown in box A. Additional sample information was available at the NCI website (http://ccr.cancer.gov/tech_initiatives/tarp).
(2.31 MB PDF)

Figure S2 Three pathways enriched in various cancer types. (A) glycolysis pathway, (B) cell cycle checkpoint II pathway and (C) plk3 pathway. Dark red, overexpressed in tumors (p<0.01); light red, overexpressed in tumors (p>0.01); dark green, underexpressed in tumors (p<0.01); light green, underexpressed in tumors (p>0.01); white, missing data.
(0.61 MB PDF)

Figure S3 Hierarchical clustering of gene-expression profiles for 187 common cancer genes in datasets 21–38. Normal tissues were marked black and tumor tissues were marked red. The accuracy of classification is, on average, 92.64% ranging from 78% to 100%.
(2.19 MB PDF)

Figure S4 Hierarchical clustering of gene-expression profiles in dataset 39 using 28 common cancer genes confirmed by the QRT-PCR analysis with fold change >3 and consistency >60%. Samples are also clustered into three groups: tumor group I composing of 123 tumors and 18 normal tissues, tumor group II composing of 46 tumor and 24 normal tissues, and normal group composing of 39 normal tissues and 11 tumors. The accuracy of classification is 80%.
(0.15 MB PDF)

Table S1 Datasets in the integrated gene-expression analyses
(0.17 MB DOC)

Table S2 Functional categories of common up- and down-regulated cancer genes
(0.07 MB DOC)

Table S3 A comparison of several signatures in independent datasets
(0.03 MB DOC)

Table S4 Oligonucleotide primers and probes used for real-time PCR Analysis
(0.05 MB DOC)
