# Supplementary figures and images for: Common Human Cancer Genes Discovered by Integrated Gene-Expression Analysis
Source: PLoS One. 2007 Nov 7;2(11):e1149. doi: 10.1371/journal.pone.0001149 (PMC2065803; doi:10.1371/journal.pone.0001149)

SPP1

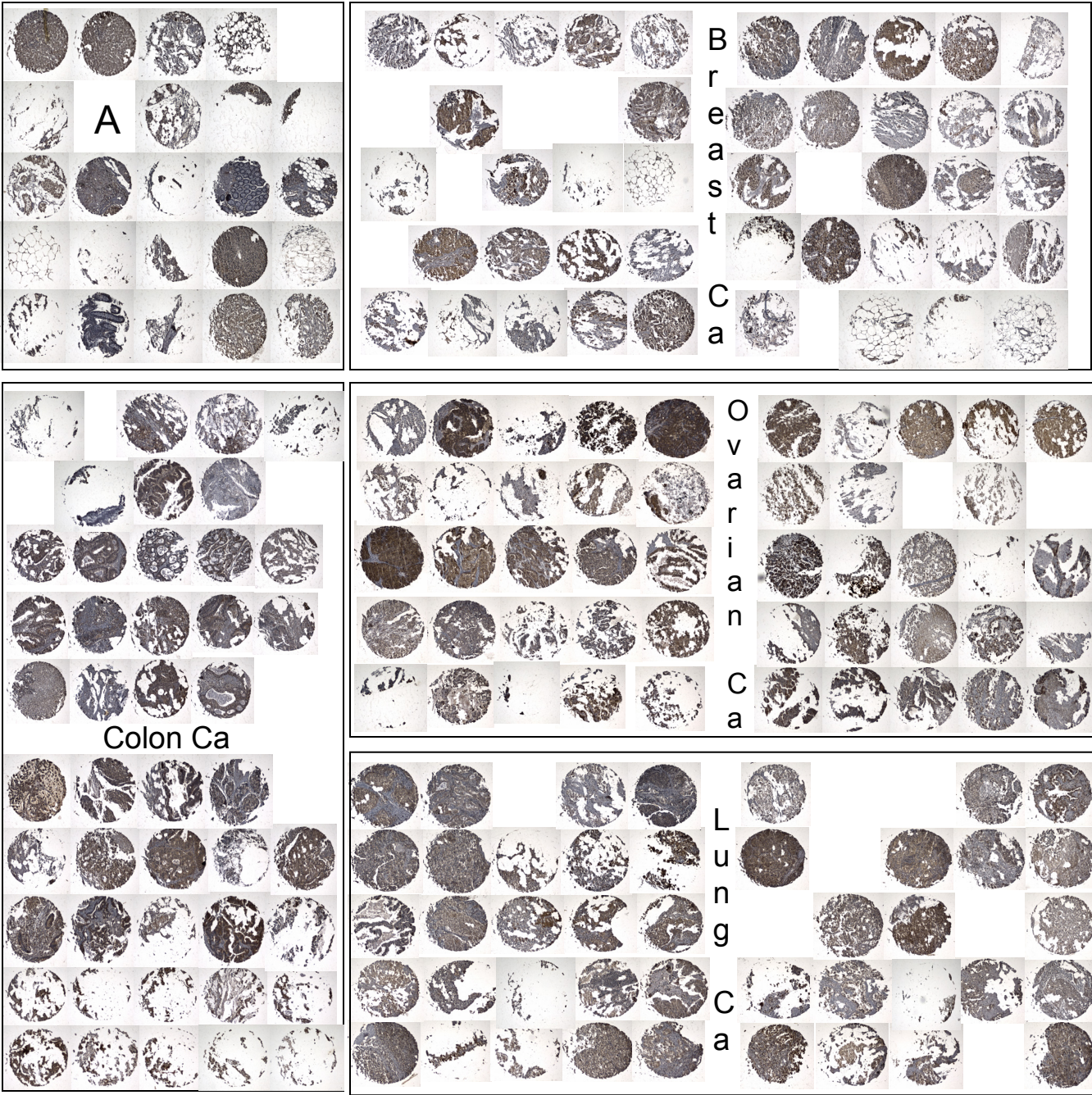

Supplement: Figure S1 — The immunostaining images of SPP1 in various cancer tissue microarray. The sections from normal tissues are shown in box A. Additional sample information was available at the NCI website (http://ccr.cancer.gov/tech_initiatives/tarp). (2.31 MB PDF) [file pone.0001149.s001.pdf]

A

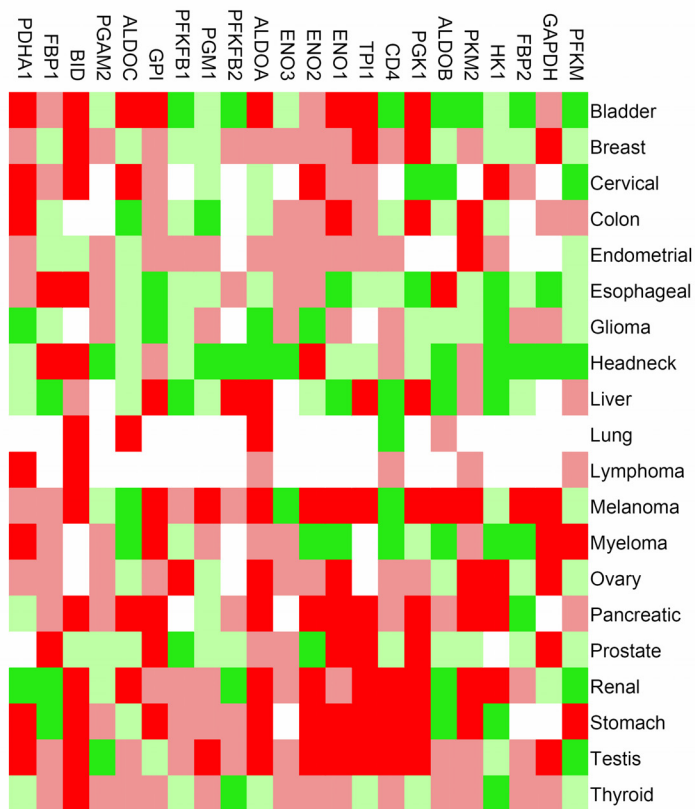

B

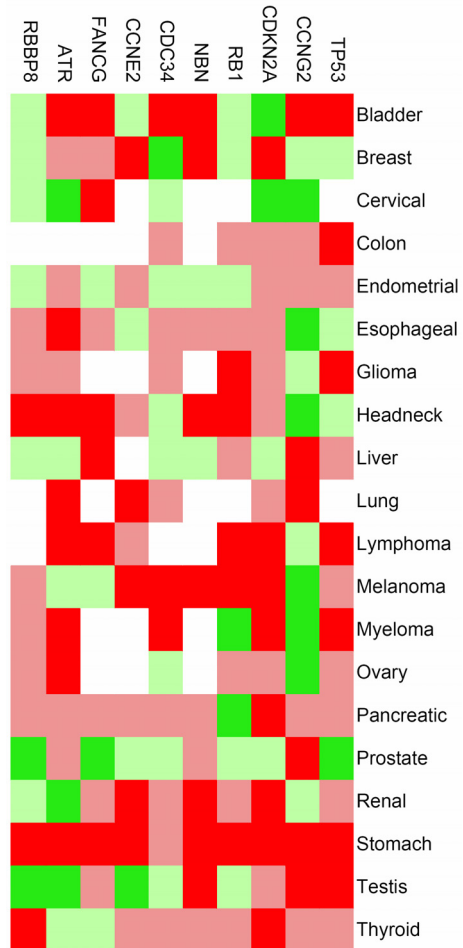

C

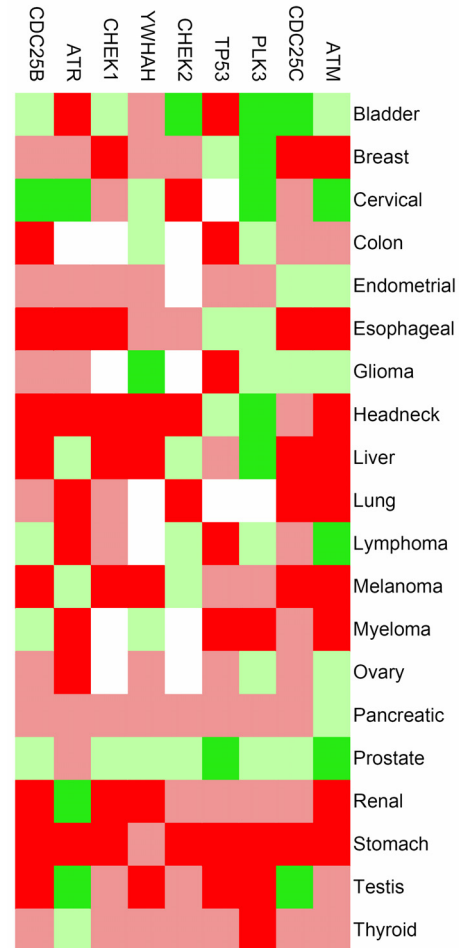

Supplement: Figure S2 — Three pathways enriched in various cancer types. (A) glycolysis pathway, (B) cell cycle checkpoint II pathway and (C) plk3 pathway. Dark red, overexpressed in tumors (p<0.01); light red, overexpressed in tumors (p>0.01); dark green, underexpressed in tumors (p<0.01); light green, underexpressed in tumors (p>0.01); white, missing data. (0.61 MB PDF) [file pone.0001149.s002.pdf]
