# Supplementary material for: Common Human Cancer Genes Discovered by Integrated Gene-Expression Analysis
Source: PLoS One. 2007 Nov 7;2(11):e1149. doi: 10.1371/journal.pone.0001149 (PMC2065803; doi:10.1371/journal.pone.0001149)

Dataset 21 (Colon cancer)

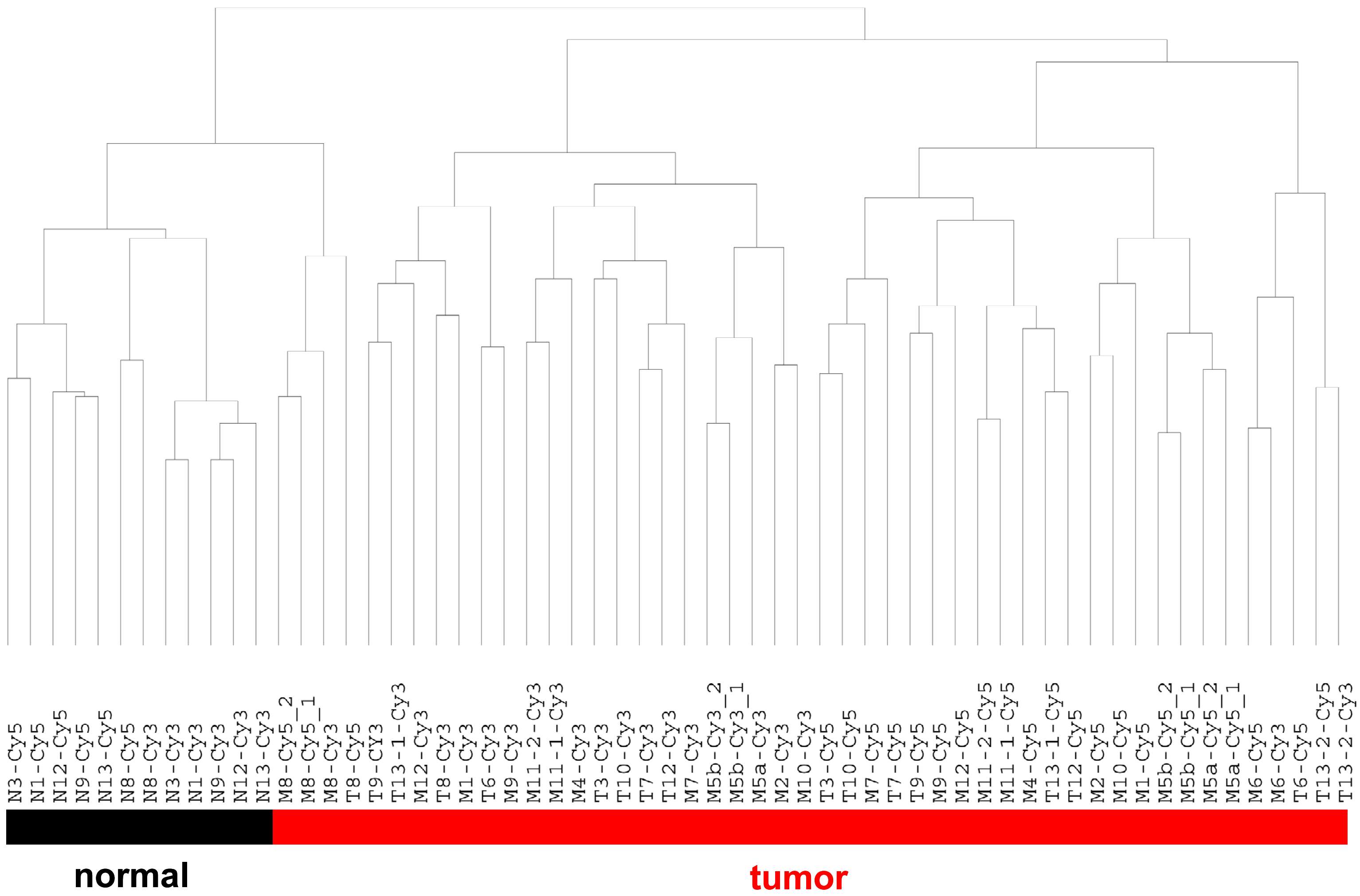

Dataset 22 (Esophageal adenocarcinomas)

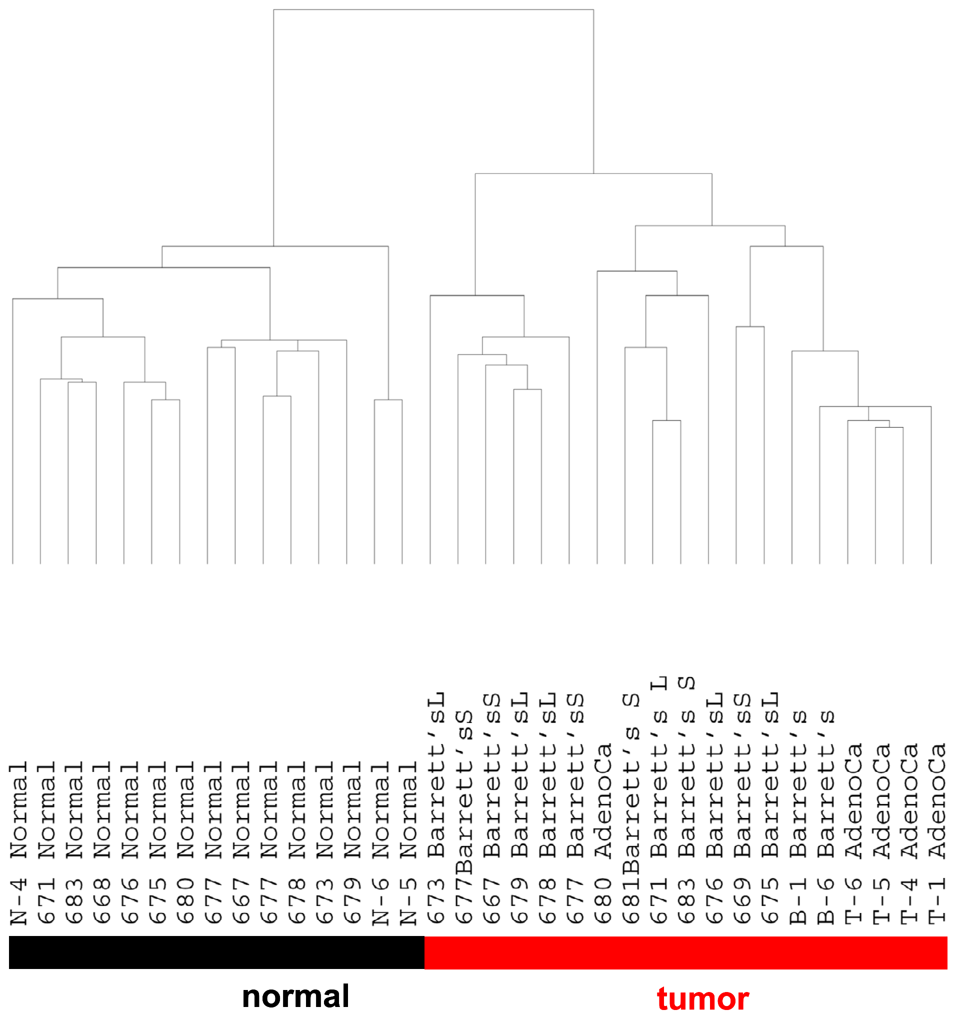

Dataset 27 (Lung Cancer)

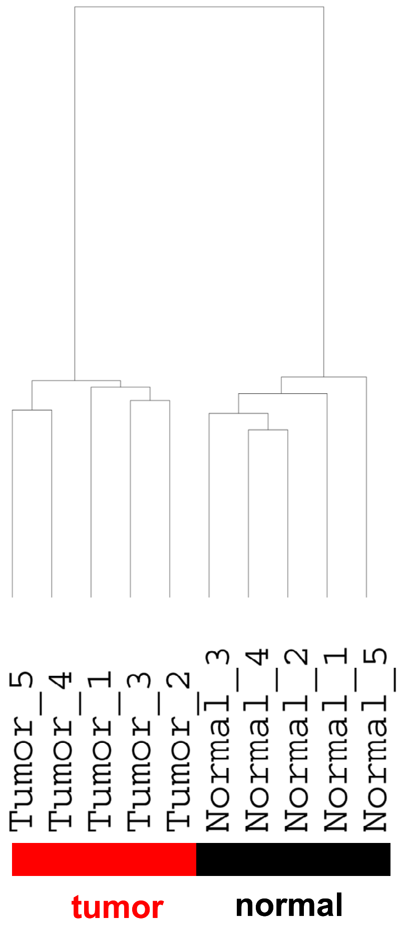

Dataset 23 (Gastric cancer)

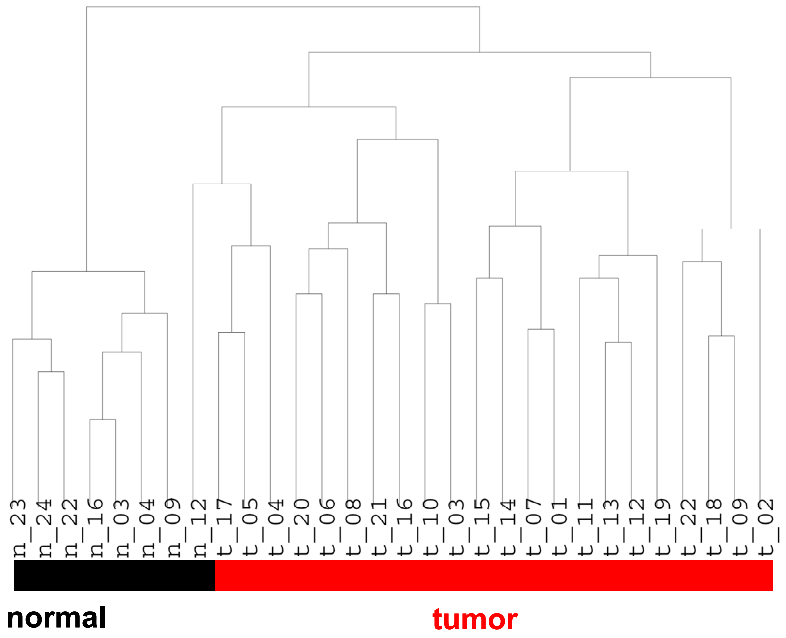

Dataset 28 (Lung cancer)

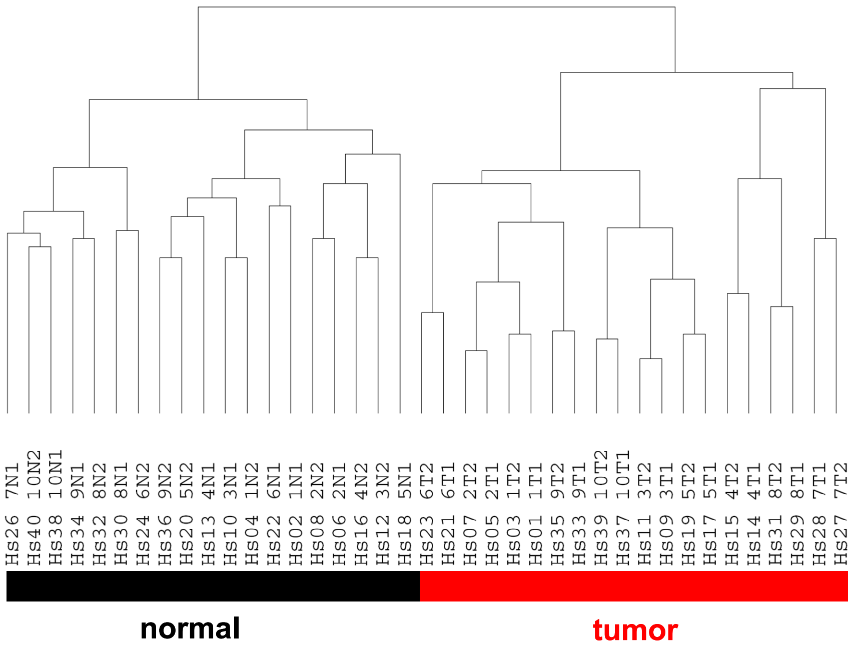

Dataset 24 (Glioblastoma)

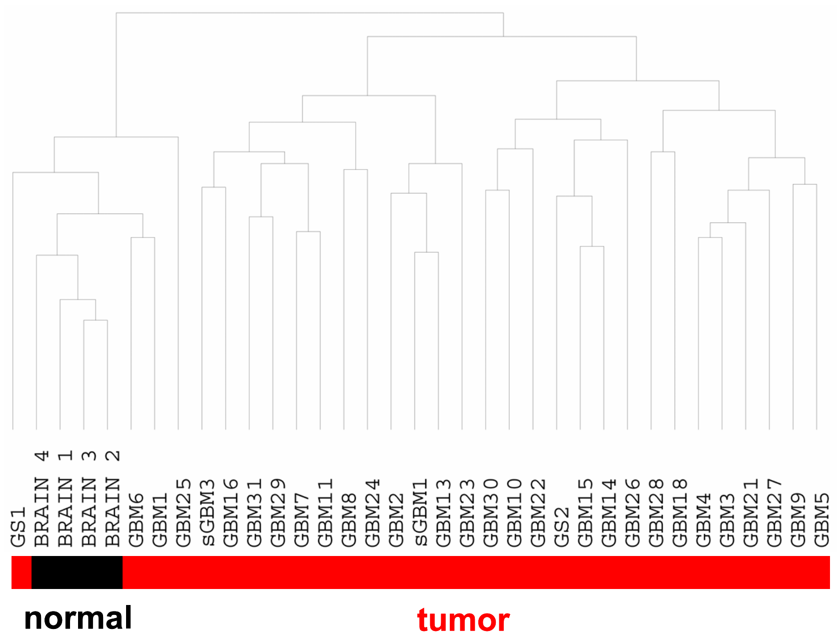

Dataset 33 (Testicular germ cell tumor)

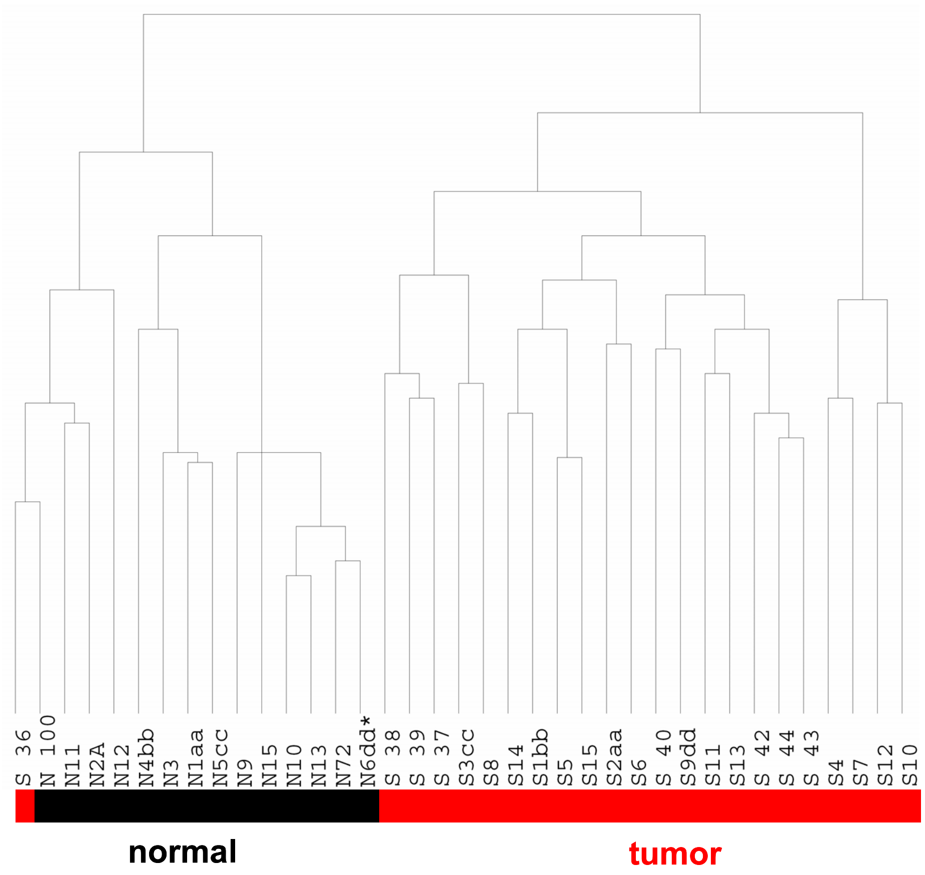

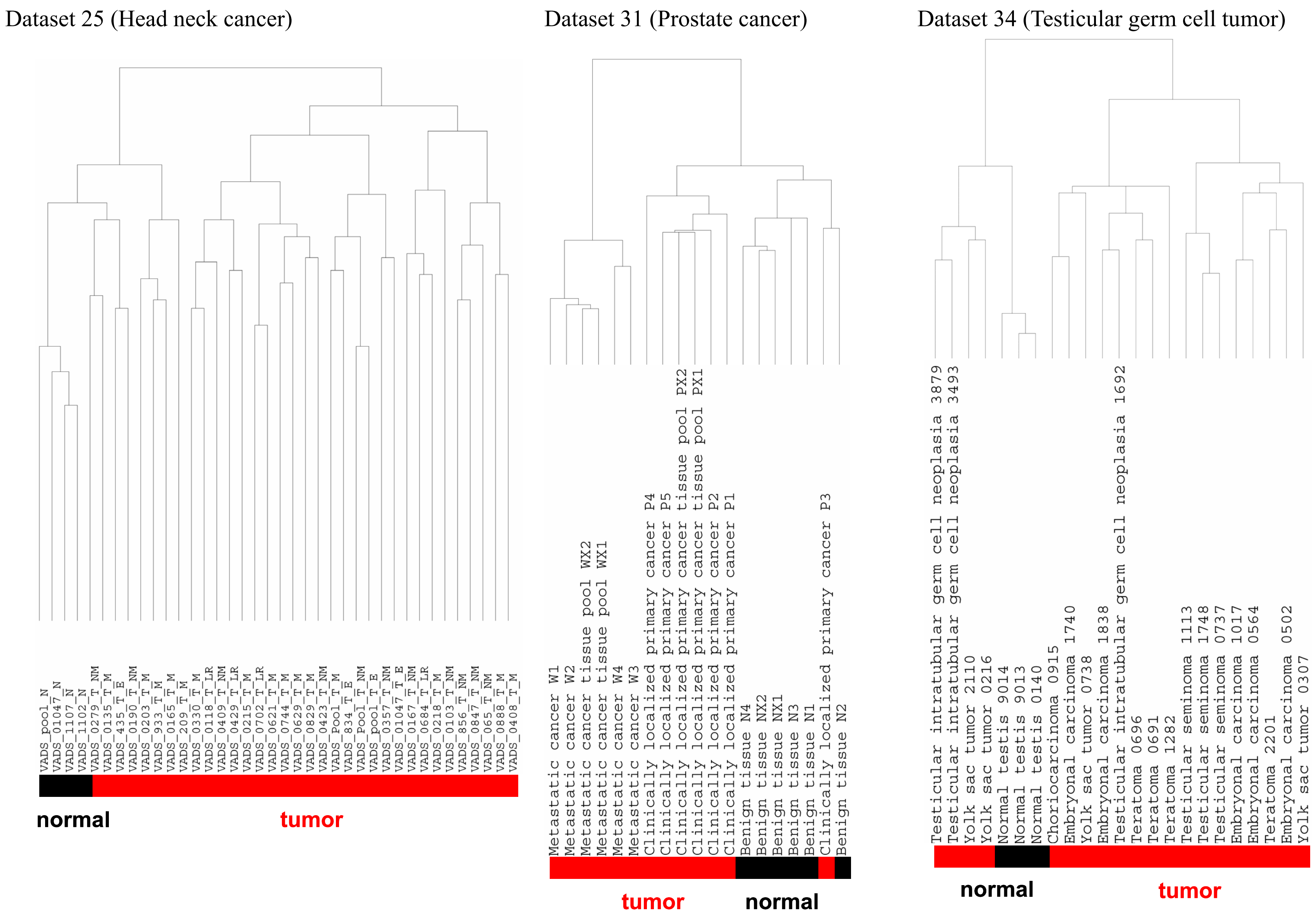

Dataset 25 (Head neck cancer)
VADS_pool_N
VADS_01047_N
VADS_1107_N
VADS_1102_N
VADS_0279_T_NM
VADS_0135_T_M
VADS_435_T_E
VADS_0190_T_NM
VADS_0203_T_M
VADS_933_T_M
VADS_0165_T_M
VADS_209_T_M
VADS_0330_T_M
VADS_0118_T_LR
VADS_0409_T_NM
VADS_0429_T_LR
VADS_0215_T_M
VADS_0702_T_LR
VADS_0621_T_M
VADS_0744_T_M
VADS_0629_T_M
VADS_0829_T_M
VADS_0423_T_NM
VADS_Pool_T_M
VADS_834_T_E
VADS_Pool_T_NM
VADS_pool_T_E
VADS_0357_T_NM
VADS_01047_T_E
VADS_0167_T_NM
VADS_0684_T_LR
VADS_0218_T_M
VADS_0103_T_NM
VADS_856_T_NM
VADS_0847_T_NM
VADS_065_T_NM
VADS_0888_T_M
VADS_0408_T_M
normal
tumor
Dataset 31 (Prostate cancer)
Metastatic cancer W1
Metastatic cancer W2
Metastatic cancer tissue pool WX2
Metastatic cancer tissue pool WX1
Metastatic cancer W4
Metastatic cancer W3
Clinically localized primary cancer P4
Clinically localized primary cancer P5
Clinically localized primary cancer tissue pool PX2
Clinically localized primary cancer tissue pool PX1
Clinically localized primary cancer P2
Clinically localized primary cancer P1
Benign tissue N4
Benign tissue NX2
Benign tissue NX1
Benign tissue N3
Benign tissue N1
Clinically localized primary cancer P3
Benign tissue N2
tumor
normal
Dataset 34 (Testicular germ cell tumor)
Testicular intratubular germ cell neoplasia 3879
Testicular intratubular germ cell neoplasia 3493
Yolk sac tumor 2110
Yolk sac tumor 0216
Normal testis 9014
Normal testis 9013
Normal testis 0140
Choriocarcinoma 0915
Embryonal carcinoma 1740
Yolk sac tumor 0738
Embryonal carcinoma 1838
Testicular intratubular germ cell neoplasia 1692
Teratoma 0696
Teratoma 0691
Teratoma 1282
Testicular seminoma 1113
Testicular seminoma 1748
Testicular seminoma 0737
Embryonal carcinoma 1017
Embryonal carcinoma 0564
Teratoma 2201
Embryonal carcinoma 0502
Yolk sac tumor 0307
normal
tumor

Dataset 26 (Lung Cancer)

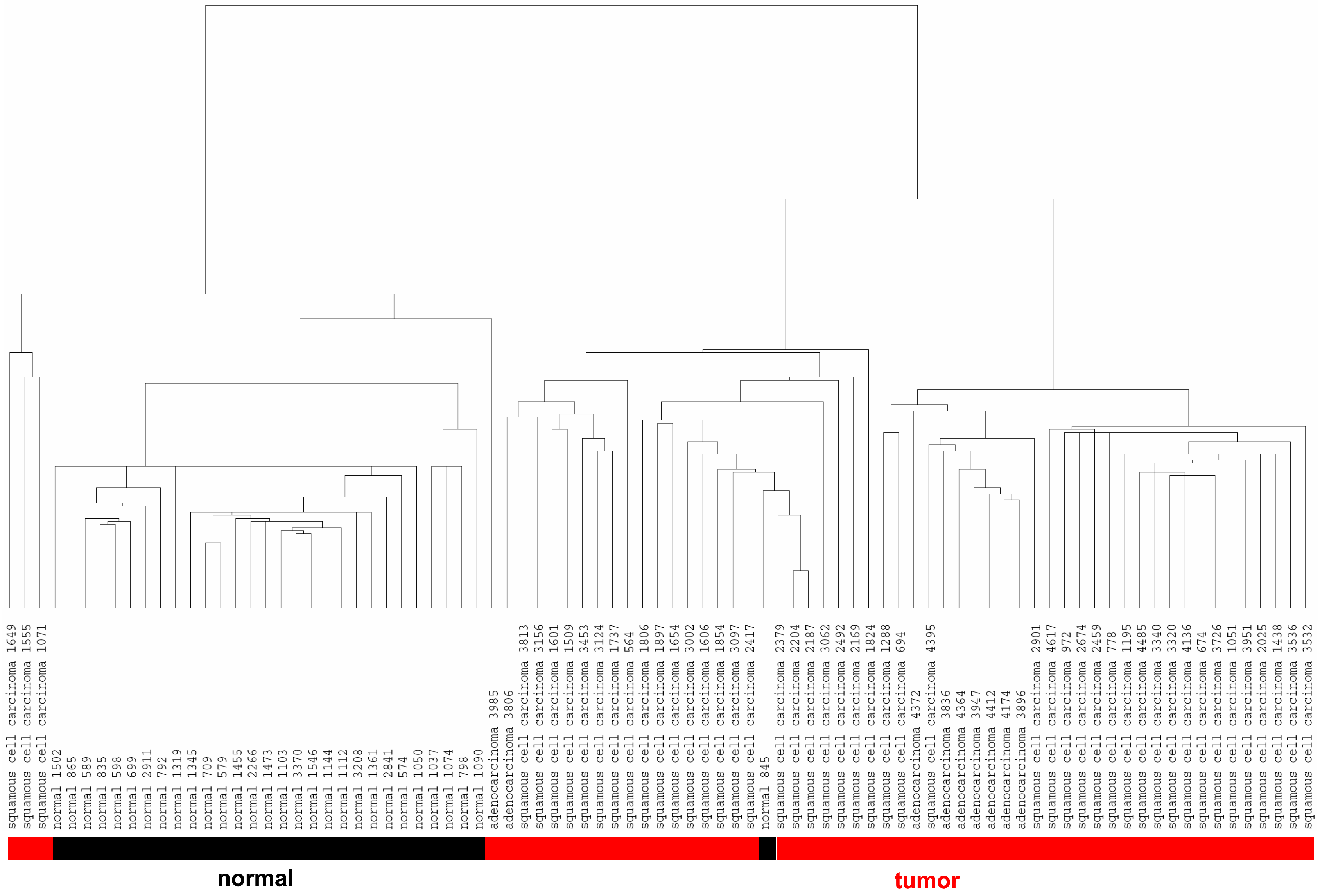

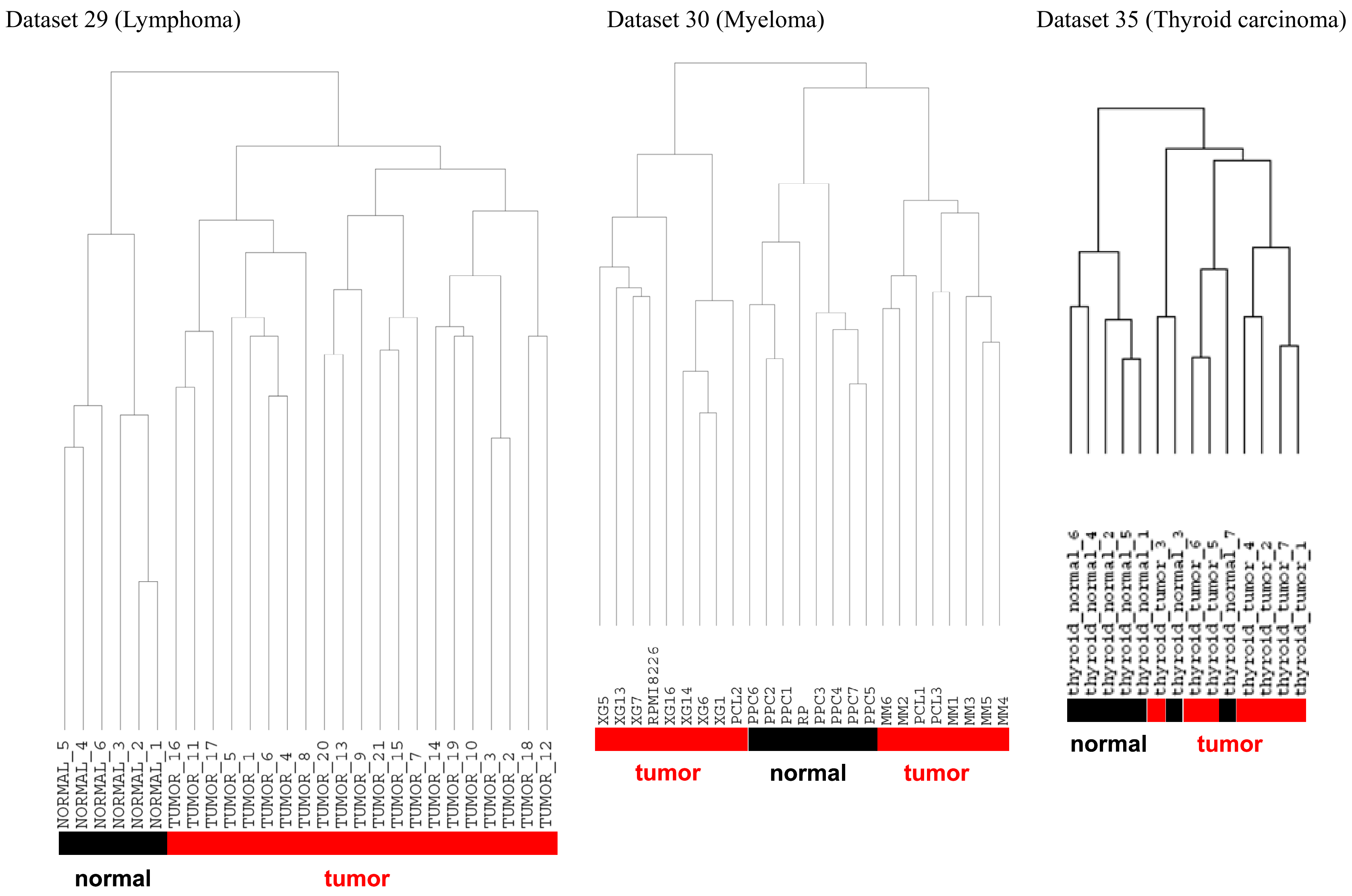

Dataset 29 (Lymphoma)
NORMAL_5
NORMAL_4
NORMAL_6
NORMAL_3
NORMAL_2
NORMAL_1
TUMOR_16
TUMOR_11
TUMOR_17
TUMOR_5
TUMOR_1
TUMOR_6
TUMOR_4
TUMOR_8
TUMOR_20
TUMOR_13
TUMOR_9
TUMOR_21
TUMOR_15
TUMOR_7
TUMOR_14
TUMOR_19
TUMOR_10
TUMOR_3
TUMOR_2
TUMOR_18
TUMOR_12
normal
tumor
Dataset 30 (Myeloma)
XG5
XG13
XG7
RPMI8226
XG16
XG14
XG6
XG1
PCL2
PPC6
PPC2
PPC1
RP
PPC3
PPC4
PPC7
PPC5
MM6
MM2
PCL1
PCL3
MM1
MM3
MM5
MM4
tumor
normal
tumor
Dataset 35 (Thyroid carcinoma)
thyroid_normal_6
thyroid_normal_4
thyroid_normal_2
thyroid_normal_5
thyroid_normal_1
thyroid_tumor_3
thyroid_normal_3
thyroid_tumor_6
thyroid_tumor_5
thyroid_normal_7
thyroid_tumor_4
thyroid_tumor_2
thyroid_tumor_7
thyroid_tumor_1
normal
tumor

Dataset 32 (Prostate cancer)

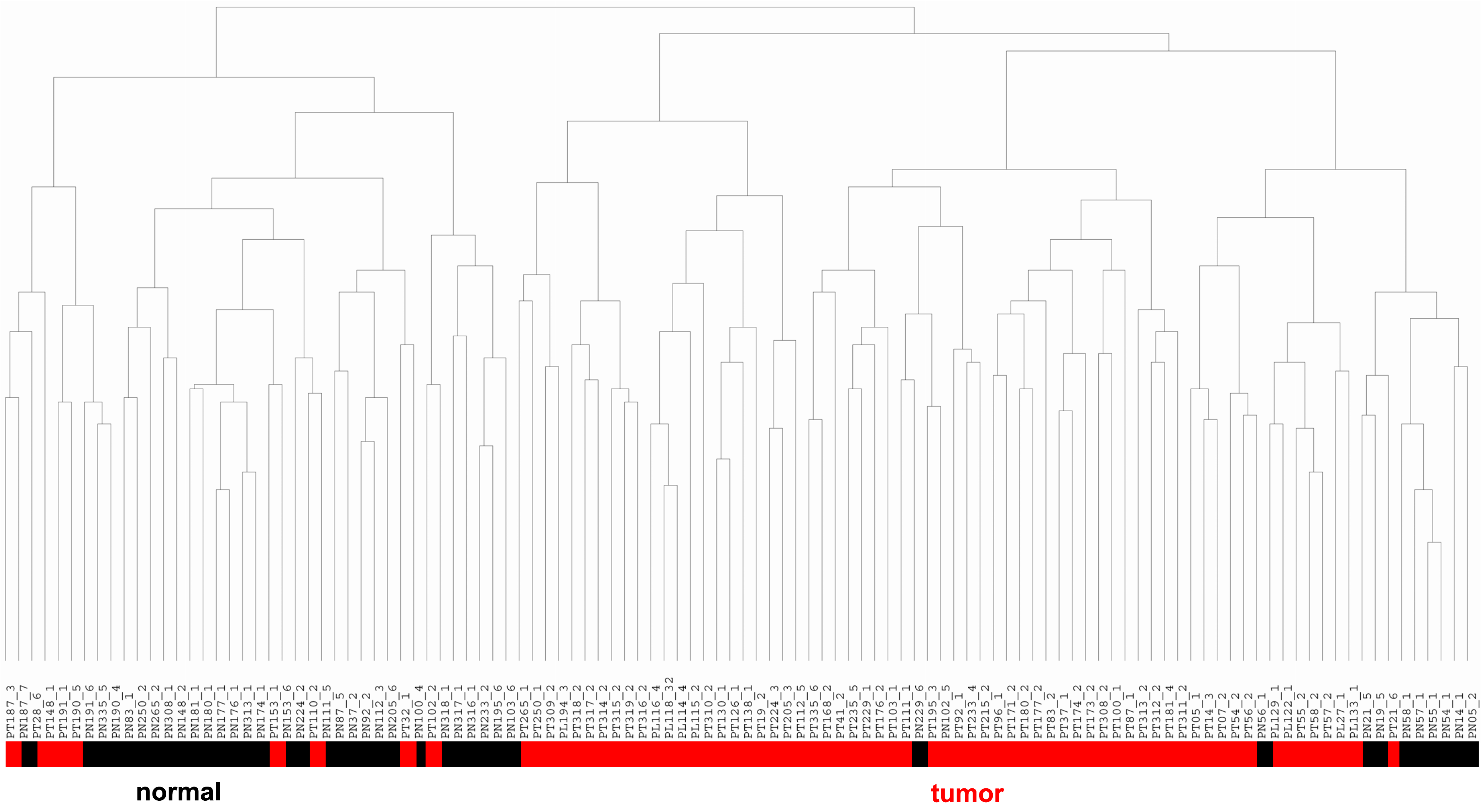

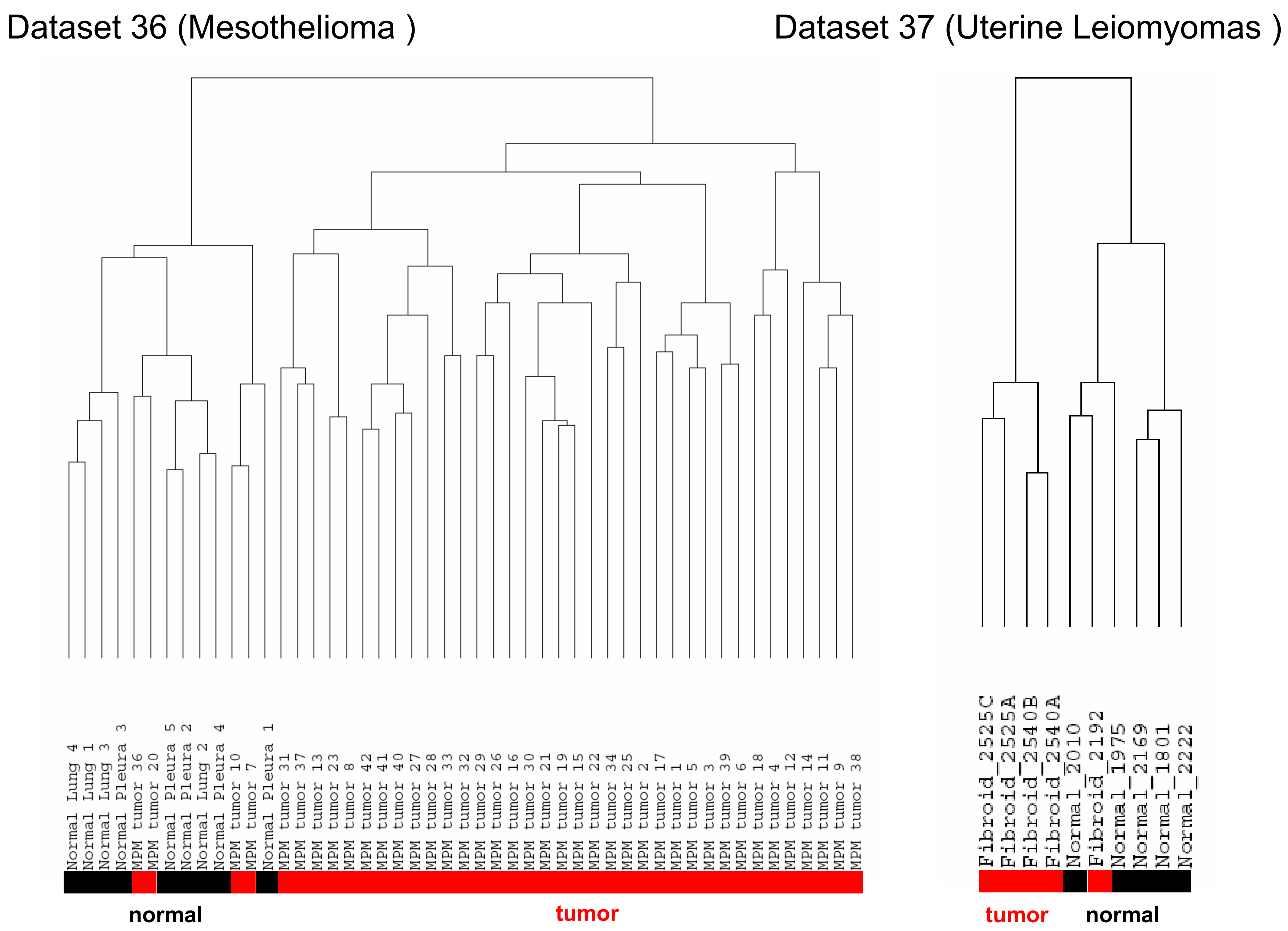
Dataset 36 (Mesothelioma)
Dataset 37 (Uterine Leiomyomas)
Normal Lung 4
Normal Lung 1
Normal Lung 3
Normal Pleura 3
MPM tumor 36
MPM tumor 20
Normal Pleura 5
Normal Pleura 2
Normal Lung 2
Normal Pleura 4
MPM tumor 10
MPM tumor 7
Normal Pleura 1
MPM tumor 31
MPM tumor 37
MPM tumor 13
MPM tumor 23
MPM tumor 8
MPM tumor 42
MPM tumor 41
MPM tumor 40
MPM tumor 27
MPM tumor 28
MPM tumor 33
MPM tumor 32
MPM tumor 29
MPM tumor 26
MPM tumor 16
MPM tumor 30
MPM tumor 21
MPM tumor 19
MPM tumor 15
MPM tumor 22
MPM tumor 34
MPM tumor 25
MPM tumor 2
MPM tumor 17
MPM tumor 1
MPM tumor 5
MPM tumor 3
MPM tumor 39
MPM tumor 6
MPM tumor 18
MPM tumor 4
MPM tumor 12
MPM tumor 14
MPM tumor 11
MPM tumor 9
MPM tumor 38
normal
tumor
Fibroid_2525C
Fibroid_2525A
Fibroid_2540B
Fibroid_2540A
Normal_2010
Fibroid_2192
Normal_1975
Normal_2169
Normal_1801
Normal_2222
tumor
normal

Dataset 38 (Soft Tissue Sarcoma)

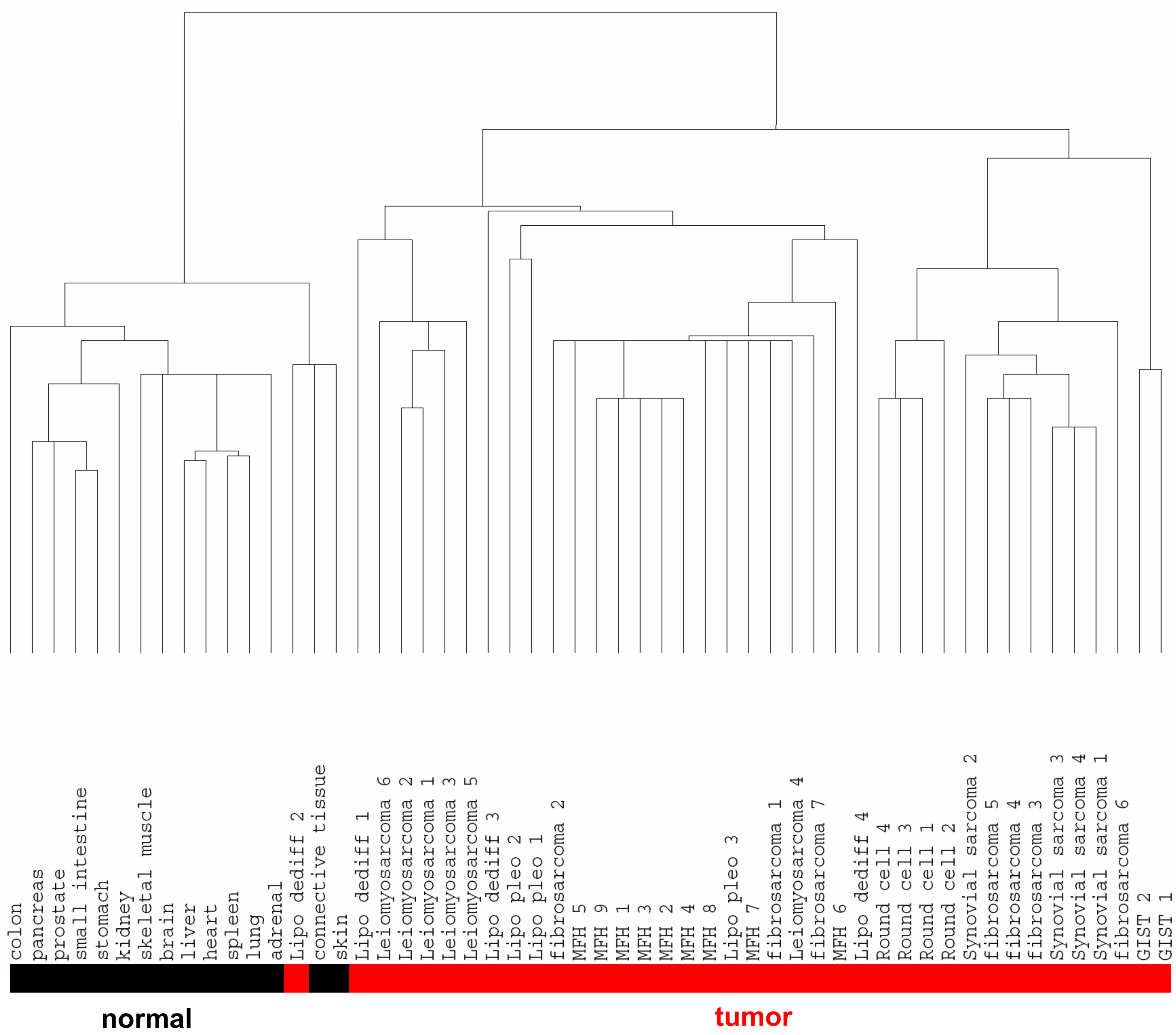

Supplement: Figure S3 — Hierarchical clustering of gene-expression profiles for 187 common cancer genes in datasets 21–38. Normal tissues were marked black and tumor tissues were marked red. The accuracy of classification is, on average, 92.64% ranging from 78% to 100%. (2.19 MB PDF) [file pone.0001149.s003.pdf]
