# Supplementary material for: Common Human Cancer Genes Discovered by Integrated Gene-Expression Analysis
Source: PLoS One. 2007 Nov 7;2(11):e1149. doi: 10.1371/journal.pone.0001149 (PMC2065803; doi:10.1371/journal.pone.0001149)
Supplement: Figure S4 — Hierarchical clustering of gene-expression profiles in dataset 39 using 28 common cancer genes confirmed by the QRT-PCR analysis with fold change >3 and consistency >60%. Samples are also clustered into three groups: tumor group I composing of 123 tumors and 18 normal tissues, tumor group II composing of 46 tumor and 24 normal tissues, and normal group composing of 39 normal tissues and 11 tumors. The accuracy of classification is 80%. (0.15 MB PDF) [file pone.0001149.s004.pdf]

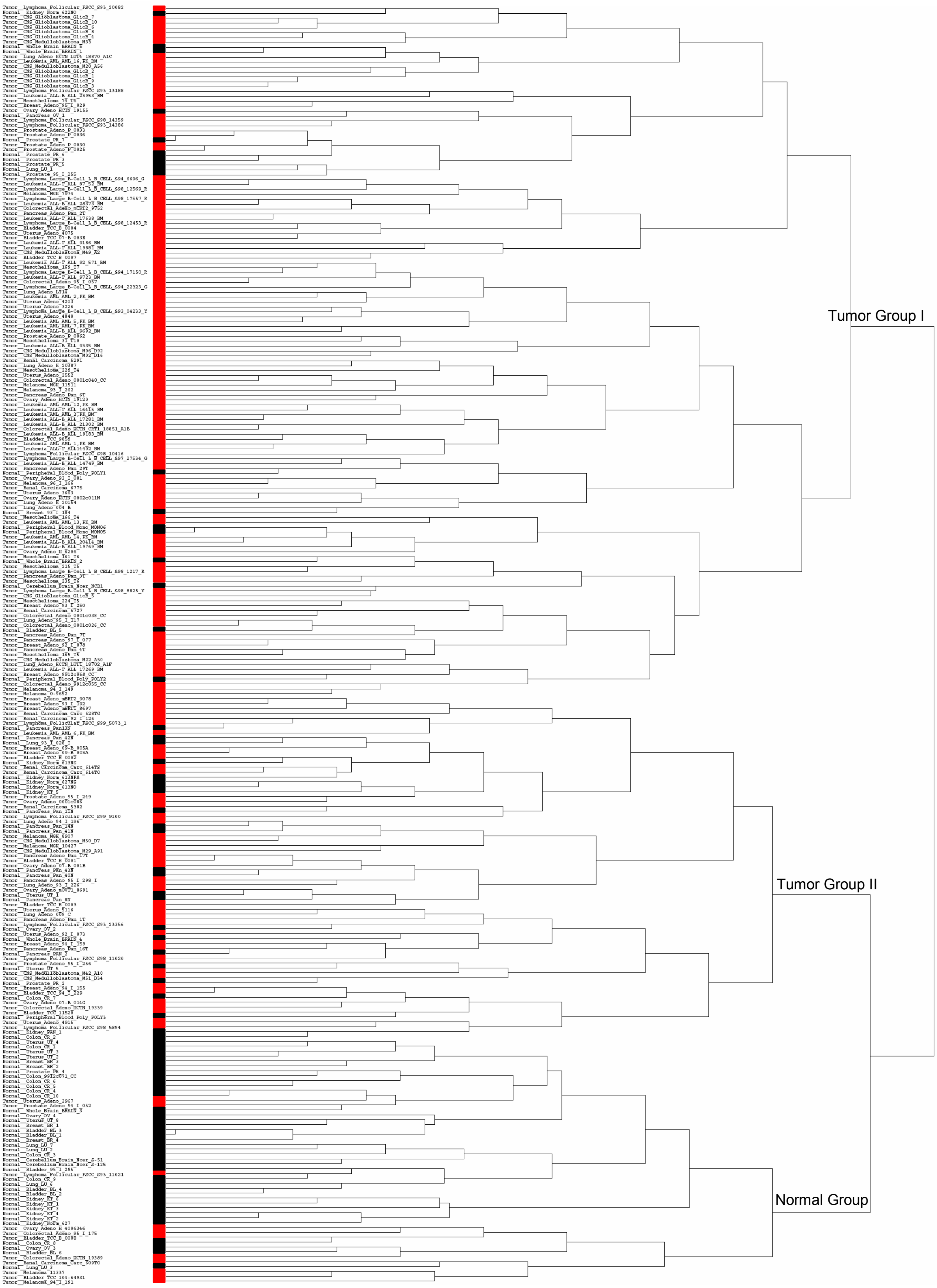
Tumor Group I
Tumor Group II
Normal Group
