# Supplementary material for: Common Human Cancer Genes Discovered by Integrated Gene-Expression Analysis
Source: PLoS One. 2007 Nov 7;2(11):e1149. doi: 10.1371/journal.pone.0001149 (PMC2065803; doi:10.1371/journal.pone.0001149)
Supplement: Table S1 — Datasets in the integrated gene-expression analyses (0.17 MB DOC) [file pone.0001149.s005.doc]

**Table S1** Datasets in the integrated gene-expression analyses

| **Dataset** | **Cancer type** | **NN** | **NT** | **GEO number/ Webpage** | **Reference** |
| --- | --- | --- | --- | --- | --- |
| 1 | Bladder cancer | 9 | 41 | GSE3167 | [1] |
| 2 | Breast cancer | 7 | 40 | GSE3744 | [2] |
| 3 | Cervical cancer | 8 | 25 | GSE527 | [3] |
| 4 | Colon cancer | 18 | 18 | http://microarray.princeton.edu/oncology | [4] |
| 5 | Endometrial cancer | 7 | 35 | http://home.ccr.cancer.gov/risingerdata1102/ | [5] |
| 6 | Esophageal adenocarcinomas | 8 | 8 | GSE1420 | [6] |
| 7 | Gastric Cancer | 23 | 89 | GSE2701 | [7] |
| 8 | Glioma | 6 | 45 | http://gedp.nci.nih.gov/ | [8] |
| 9 | Head neck squamous cell cancer | 13 | 41 | http://www.gaffney.umn.edu/suppl/ | [9] |
| 10 | Liver cancer | 76 | 104 | GSE3500 | [10] |
| 11 | Lung cancer | 17 | 52 | http://www.broad.mit.edu/mpr/lung/ | [11] |
| 12 | Lymphoma | 15 | 50 | GDS75 | [12] |
| 13 | Melanoma | 25 | 45 | GSE3189 | [13] |
| 14 | Myeloma | 30 | 74 | http://lambertlab.uams.edu/software/ | [14] |
| 15 | Ovarian cancer | 4 | 27 | http://www.gnf.org/cancer/ovary/ | [15] |
| 16 | Pancreatic adenocarcinoma | 5 | 16 | GSE3654 | [16] |
| 17 | Prostate cancer | 50 | 52 | [http://www-genome.wi.mit.edu/MPR/prostate](http://www.sciencedirect.com/science?_ob=RedirectURL&_method=externObjLink&_locator=url&_cdi=7133&_plusSign=%2B&_targetURL=http%253A%252F%252Fwww-genome.wi.mit.edu%252FMPR%252Fprostate) | [17] |
| 18 | Renal cell carcinoma | 8 | 9 | GSE781 | [18] |
| 19 | Testicular germ cell tumors | 6 | 101 | GSE3218 | [41] |
| 20 | Thyroid carcinoma | 8 | 8 | [http://thinker.med.ohio-state.edu](http://thinker.med.ohio-state.edu/) | [19] |
| 21 | Colon cancer | 12 | 48 | GSE3964 | [42] |
| 22 | Esophageal adenocarcinomas | 15 | 19 | GSE6059 | [43] |
| 23 | Gastric Cancer | 8 | 22 | GSE2685 | [44] |
| 24 | Glioblastoma | 4 | 31 | GSE2223 | [45] |
| 25 | Head neck hypopharyngeal cancer | 4 | 34 | GSE2379 | [46] |
| 26 | Lung cancer | 30 | 57 | GSE2088 | - |
| 27 | Lung cancer | 5 | 5 | GSE3268 | [47] |
| 28 | Lung cancer | 19 | 20 | GSE2514 | [48] |
| 29 | Lymphoma | 6 | 21 | GSE3827 | [49] |
| 30 | Myeloma | 8 | 17 | <http://www.u475.montp.inserm.fr/BK/SupplementalData.htm> | [50] |
| 31 | Prostate cancer | 6 | 13 | GSE3325 | [51] |
| 32 | Prostate cancer | 41 | 71 | GSE3933 | [52] |
| 33 | Testicular germ cell tumors | 14 | 23 | <http://microarray-pubs.stanford.edu/es_cells_2/download.shtml> | [38] |
| 34 | Testicular germ cell tumors | 3 | 20 | GSE1818 | [53] |
| 35 | Thyroid carcinoma | 7 | 7 | GSE3678 | - |
| 36 | Mesothelioma | 9 | 40 | GSE2549 | [54] |
| 37 | Uterine Leiomyomas | 5 | 5 | GSE593 | [55] |
| 38 | Soft Tissue Sarcoma | 15 | 39 | GSE2719 | [56] |
| 39 | Multiclass cancer | 80 | 180 | ftp://ftp.broad.mit.edu/pub/gcm_files/ | [57] |

NN: number of normal samples. NT: number of tumor samples.
