# Supplementary material for: Common Human Cancer Genes Discovered by Integrated Gene-Expression Analysis
Source: PLoS One. 2007 Nov 7;2(11):e1149. doi: 10.1371/journal.pone.0001149 (PMC2065803; doi:10.1371/journal.pone.0001149)
Supplement: Table S2 — Functional categories of common up- and down-regulated cancer genes (0.07 MB DOC) [file pone.0001149.s006.doc]

**Table S2** Functional categories of common up- and down-regulated cancer genes

| **GO term** | **Common up- and down-regulated genes** | **Percentage** |
| --- | --- | --- |
| cellular macromolecule metabolism | PTP4A3 BFAR GLT25D1 PSMB4 FBXO3 RPN2 TTK PDIA6 APOL2 USP2 ADAM10 PSMA4 FKBP11 PPIH STAT1 EPRS MSRA EIF2C2 HSPE1 PLOD3 PINK1 WHSC1 TUBAL3 PSMB8 PSMA7 TAPBP EIF2S2 MAGI1 NAP1L1 CDC14B NPM1 ERBB4 CCT5 PSMB2 FMNL3 FBXO32 ADAM12 DTX3L WASF3 FKBP10 PPP2R1B TTL | 39.25 |
| protein metabolism | PTP4A3 BFAR PSMB4 FBXO3 RPN2 TTK PDIA6 APOL2 USP2 ADAM10 PSMA4 FKBP11 PPIH STAT1 EPRS MSRA EIF2C2 HSPE1 PLOD3 PINK1 WHSC1 TUBAL3 PSMB8 PSMA7 TAPBP EIF2S2 MAGI1 NAP1L1 CDC14B NPM1 ERBB4 CCT5 PSMB2 FMNL3 FBXO32 ADAM12 DTX3L WASF3 FKBP10 PPP2R1B TTL | 38.32 |
| biopolymer metabolism | PTP4A3 BFAR GLT25D1 PSMB4 FBXO3 RPN2 TTK NCL USP2 ADAM10 PSMA4 PPIH LSM4 SNRPB NONO STAT1 FEN1 EPRS HNRPU MSRA PLOD3 PINK1 WHSC1 DNMT1 PSMB8 DBR1 PSMA7 NAP1L1 CDC14B ERBB4 DDX39 CBX3 PSMB2 FBXO32 DTX3L CIDEA PPP2R1B TTL | 35.51 |
| nucleobase, nucleoside, nucleotide and nucleic acid metabolism | FOXP1 LDHD NCL RORA ALDH6A1 NR3C2 HIF3A PPIH LSM4 SNRPB NONO STAT1 FEN1 EPRS ILF3 NME1 HNRPU WHSC1 DNMT1 DBR1 ACLY ZBTB12 NAP1L1 NPM1 HLF DDX39 CBX3 CIDEA E2F3 IQGAP3 PPP2R1B | 28.97 |
| transport | SNX10 KDELR3 TRPM3 SLC24A3 LDHD TAP1 PDIA6 APOL2 ADAM10 SFXN1 PPIH NOX4 STAT1 KDELR2 TUBAL3 FTL TAPBP GULP1 TNPO1 NPM1 SLC38A1 DDX39 KCNAB1 AP1S1 FMO4 IPO9 | 24.3 |
| regulation of cellular metabolism | FOXP1 RORA NR3C2 HIF3A NONO STAT1 ILF3 WHSC1 DNMT1 ZBTB12 NPM1 HLF CBX3 E2F3 PPP2R1B | 14.02 |
| intracellular transport, cellular localization and establishment of cellular localization | KDELR3 ADAM10 PPIH STAT1 KDELR2 TUBAL3 TAPBP TNPO1 NPM1 DDX39 AP1S1 IPO9 | 11.21 |
| programmed cell death | BFAR CARD4 SPP1 SNCA BAX STAT1 CLU GULP1 BID CIDEA PPP2R1B | 10.28 |
| cellular biosynthesis | GLT25D1 LDHD RPN2 EPRS NME1 EIF2C2 EFCBP1 EIF2S2 FMNL3 PPP2R1B | 9.35 |
| cellular catabolism | EPHX2 PGK1 PSMB4 USP2 PSMA4 PSMB8 PSMA7 ACLY PSMB2 CIDEA | 9.35 |
| protein transport and establishment of protein localization | SNX10 KDELR3 TAP1 PPIH STAT1 KDELR2 TNPO1 NPM1 AP1S1 IPO9 | 9.35 |
| negative regulation of cellular physiological process | BFAR RECK SPP1 SNCA BAX ILF3 NME1 DNMT1 NPM1 PPP2R1B | 9.35 |
| organelle organization and biogenesis | PRC1 TTK DMD GNL2 TUBAL3 NAP1L1 NPM1 CBX3 FMNL3 WASF3 | 9.35 |
| regulation of programmed cell death | BFAR CARD4 SPP1 SNCA BAX STAT1 BID CIDEA PPP2R1B | 8.41 |
| intracellular signaling cascade | SNX10 CARD4 STAT1 FEN1 SHANK2 PINK1 TMEPAI IQGAP3 PPP2R1B | 8.41 |
| positive regulation of cellular physiological process | TTK SPP1 BAX ILF3 BID NAP1L1 CIDEA TNFSF13B PPP2R1B | 8.41 |
| phosphorus metabolism | PTP4A3 PGK1 TTK ADAM10 STAT1 PINK1 CDC14B ERBB4 PPP2R1B | 8.41 |
| regulation of cell cycle | TTK RECK BAX STAT1 NME1 CCNB2 E2F3 PPP2R1B | 7.48 |
| immune response | TAP1 APOL2 SPP1 CLU PSMB8 TAPBP HLA-F TNFSF13B | 7.48 |
| macromolecule catabolism | PGK1 PSMB4 USP2 PSMA4 PSMB8 PSMA7 PSMB2 | 6.54 |
| macromolecule biosynthesis | GLT25D1 RPN2 EPRS EIF2C2 EIF2S2 FMNL3 PPP2R1B | 6.54 |
| lipid metabolism | GLT25D1 PPAP2B APOL2 CLU ACLY PLIN PPP2R1B | 6.54 |
| regulation of cell proliferation | TTK SPP1 NME1 NAP1L1 NPM1 TNFSF13B | 5.61 |
| generation of precursor metabolites and energy | PGK1 LDHD PDIA6 NOX4 ACLY FMO4 | 5.61 |
| cell surface receptor linked signal transduction | ADAM10 WISP1 MAGI1 ERBB4 ADAM12 PPP2R1B | 5.61 |
| vesicle-mediated transport | KDELR3 KDELR2 TAPBP GULP1 AP1S1 | 4.67 |
| ion transport | TRPM3 SLC24A3 SFXN1 FTL KCNAB1 | 4.67 |
| organic acid metabolism | ALDH6A1 EPRS MSRA ACLY PPP2R1B | 4.67 |
| amino acid and derivative metabolism | ALDH6A1 EPRS MSRA PPP2R1B | 3.74 |
| cellular lipid metabolism | GLT25D1 APOL2 ACLY PPP2R1B | 3.74 |
| electron transport | LDHD PDIA6 NOX4 FMO4 | 3.74 |
| carbohydrate metabolism | GLT25D1 PGK1 DHRS4 ACLY | 3.74 |
| M phase | PRC1 TTK ILF3 CCNB2 | 3.74 |
| amine metabolism | ALDH6A1 EPRS MSRA PPP2R1B | 3.74 |
| response to pest, pathogen or parasite | APOL2 SPP1 STAT1 CLU | 3.74 |
| cell motility | PPAP2B ARPC1B SPP1 PALM | 3.74 |
| mitotic cell cycle | PRC1 TTK CCNB2 | 2.8 |
| negative regulation of metabolism | ILF3 DNMT1 PPP2R1B | 2.8 |
| regulation of signal transduction | CARD4 IQGAP3 PPP2R1B | 2.8 |
| secretion | KDELR3 KDELR2 TAPBP | 2.8 |
| cellular morphogenesis | WISP1 PALM PPP2R1B | 2.8 |
| secretory pathway | KDELR3 KDELR2 TAPBP | 2.8 |
| cell migration | PPAP2B SPP1 | 1.87 |
| cell ion homeostasis | NR3C2 FTL | 1.87 |
| xenobiotic metabolism | EPHX2 FMO4 | 1.87 |
| lipid biosynthesis | GLT25D1 ACLY | 1.87 |
| regulation of cell size | WISP1 PPP2R1B | 1.87 |
| DNA repair | NONO FEN1 | 1.87 |
| cytoplasm organization and biogenesis | GNL2 NPM1 | 1.87 |
| cofactor metabolism | LDHD ACLY | 1.87 |
| aromatic compound metabolism | EPHX2 ABHD6 | 1.87 |
| regulation of cell growth | WISP1 PPP2R1B | 1.87 |
