# Supplementary material for: Common Human Cancer Genes Discovered by Integrated Gene-Expression Analysis
Source: PLoS One. 2007 Nov 7;2(11):e1149. doi: 10.1371/journal.pone.0001149 (PMC2065803; doi:10.1371/journal.pone.0001149)
Supplement: Table S3 — A comparison of several signatures in independent datasets (0.03 MB DOC) [file pone.0001149.s007.doc]

**Table S3** A comparison of several signatures in independent datasets

| **Tumor types*** | **Datasets** | **Rhode's signature**** | **Xu's signature** | **Our signature** |
| --- | --- | --- | --- | --- |
| Gordon_Lung | 36 | 91.8 | 95.9 | 91.8 |
| Hoffman_Myometrium | 37 | 80.0 | 80.0 | 90.0 |
| Wachi_Lung | 27 | 100.0 | 100.0 | 100.0 |
| Yoon_Soft_Tissue | 38 | 85.2 | 96.3 | 98.2 |
| **Overall** | / | **89.3** | **93.1** | **95.0** |

* Xu et al. [21] compared their signature with Rhodes’ [22] in six independent datasets. However, two of these six datasets (datasets 13 and 18) were already used in training sets in our study and thus were excluded for the validation study. The remaining four datasets were used for the comparison of these three signatures.

** Accuracy (%) for correct classification.
