# Supplementary material for: Common Human Cancer Genes Discovered by Integrated Gene-Expression Analysis
Source: PLoS One. 2007 Nov 7;2(11):e1149. doi: 10.1371/journal.pone.0001149 (PMC2065803; doi:10.1371/journal.pone.0001149)
Supplement: Table S4 — Oligonucleotide primers and probes used for real-time PCR Analysis (0.05 MB DOC) [file pone.0001149.s008.doc]

**Table S4** Oligonucleotide primers and probes used for real-time PCR Analysis

| **Gene** | **Sense primer** | **Anti-sense primer** |
| --- | --- | --- |
| β-Actin | CAAGAGATGGCCAGGGCTGCT | TCCTTCTGCATCCTGTCGGCA |
| ABCA8 | AGAAACAAGATGAGGAAGAGAAAG | ACAAACAAAGTAGTAGGAGCAATG |
| ADAM12 | GCAGACAACCGAGAGTTTCAGAG | TCCACACTTCCACGCCTACC |
| ALDH6A1 | ATGCCAGATGCCAATAAGG | AGGACTGCTGTTGAAAGAG |
| ANK2 | CAAGAAGTCTGACAGCAATG | GATGGAGAGCGTTGAGTC |
| BID | GCTTGGGAAGAATAGAGGCAGAT | GCGAGGTGCCTGGCAATA |
| BRP44L | TTGCTATTCTTTGACATTC | TTAGTCATCTCGTGTTTG |
| CCT5 | GCTATTGAACACCTGGAC | GGCATTCACAGCAATCTC |
| CDC14B | GCCAGAGCAGATCGCCTTTG | TGTAAACCATTGCCAGATTGAGTG |
| CLU | GCTCTTCCAGGACAGGTTCTTC | CGGACGATGCGGGACTTG |
| DMD | CCTGGCTTTGAATGCTCTC | ATCTGGCGATGTTGAATGC |
| EPHX2 | ACTCCCTTCATACCAGCAAATC | TTCAGCCTCAGCCACTCC |
| ERBB4 | GGAGATAACCAGCATTGAG | TGATTAAGAGCCACTAACAC |
| FAM107A | GGGAGCGGGCAGACATTG | GCTTCTTGGGCTTGATGAG |
| FEN1 | GCCGCGTGTCCCAAAG | CCAGTCATCCCTCCTCTGTGTTG |
| HIF3A | AGCCTGGACATGAAGTTC | CGTGGATGTACTCGTAGG |
| HLF | TCCCATCTCCGAACTGTATGC | CTTGGTGTATTGCGGTTTGCT |
| KCNAB1 | TCATAACAACCAAACTCTACTG | GACCACATCCACATACTCG |
| NDRG2 | CGAGACCTGAACTTTGAG | GTTACATTCCACCACTGC |
| NME1 | GATCTTCTCAAGGAACACTAC | GTCTTCACCACATTCAGC |
| NR3C2 | CAGCCTTCAGTTCGTTCG | CAGTTCTTTGATGTAATTTGTCC |
| NUSAP1 | ACAGCAGAATCATTCAGAG | AGGAAACAGCATTCTCAGC |
| PGK1 | CTTAGAGCCAGTTGCTGTAGAACTCA | CTGGGCCTACACAGTCCTTCA |
| PLIN | GACACGGAGGGAGAGGACAC | GGCTGCTACCTCACTGAACTTG |
| PPP2R1B | ACAATTTATGATGAAGATGAGG | GTCACGAACAACAGTCTC |
| PRC1 | TCAAGAGCAAGATCAAGAACTG | CTCCTCACGCCTAGAAGC |
| RECK | CCCAGATTATTGCCCAGAGAC | AGTTCACAGCAGCCTAAGC |
| RORA | GTGGAGACAAATCATCAGGAATC | TGACGAGGACAGGAGTAGG |
| SEMA6D | TGCCTACATACTGCTGCTG | ATTGCCTTGAATAGTGATAGTCG |
| SPP1 | GTTTCGCAGACCTGACATCCA | CAACTCCTCGCTTTCCATGTG |
| SYNGR1 | GTCGTGTCTTGGCTGTTC | GGTTGCGGTTGTAGATGC |
| TCEAL2 | AAGAACAGCCACCGCACGAG | GCCTTTCCCGCACTCTCTGG |
| USP2 | CTAGAACGGGAAGACAGTAG | AACCTCGCTTAGCAATGG |
